# An NF-kB/TNF-alpha signalling feedback loop acts to coordinate tissue regeneration and macrophage behaviour in zebrafish

**DOI:** 10.1038/s41536-025-00414-1

**Published:** 2025-06-03

**Authors:** Kalliopi Arkoudi, Yue Yuan, Antonia Pia Cumine, Carlene Dyer, Elisabeth Busch-Nentwich, Isabel Bravo, Yi Feng, Robert D. Knight

**Affiliations:** 1 Centre for Craniofacial and Regenerative Biology, King’s College London, Guy’s Hospital, London, UK; 2 Carl Zeiss Microscopy GmbH, Jena, Germany; 3 William Harvey Research Institute, Barts and The London School of Medicine and Dentistry, Queen Mary University of London, Charterhouse Square, London, UK; 4 School of Biological and Behavioural Sciences, Queen Mary University of London, London, UK; 5 Centre for Inflammation Research, Institute for Regeneration and Repair, University of Edinburgh, Edinburgh, UK; 6 Cancer Research UK Scotland Centre, Institute of Genetics and Cancer, University of Edinburgh, Edinburgh, UK

**Keywords:** Cell death and immune response, Regeneration, Chemotaxis

## Abstract

Inflammatory cells are crucial regulators of infection and regeneration that actively migrate to affected tissues. NF-kB and TNF-alpha (TNFα) are master regulators of immune signalling, but their importance for immune cell migration is much less well understood. We have therefore investigated how NF-kB and TNFα regulate both macrophage function and behaviour in vivo using a zebrafish model of tissue repair. We show that NF-kB activity differentially regulates TNFα activity through Tnf receptors 1a and 1b to control macrophage responses to injury. Loss of NF-kB in macrophages results in elevated TNFα expression and results in more directional migration. Impaired NF-kB activity in macrophages perturbs tissue regeneration, causes increased proliferation, altered pro- and anti-inflammatory gene expression and delays fin regeneration. We identify a crucial role for NF-kB modulation of TNFα signaling to regulate macrophage responses to tissue injury, which are necessary for effective fin regeneration.

## Introduction

The NF-kB pathway is a master regulator of the immune system and is considered to be a critical driver of inflammation by inducing the expression of cytokines such as TNF-alpha and Interleukin 1 beta. Dysregulation of NF-kB is associated with a number of diseases, including a variety of cancers, autoimmune disorders and chronic conditions, including rheumatoid arthritis and obesity, whereas it also important for effective regeneration of tissue. Understanding how NF-kB regulates immune cell behaviour is therefore important when considering interventions to enhance regeneration or to treat disease.

Of the many NF-kB-regulated genes, the cytokine TNFα is a critical driver of inflammation and acts to induce cell death or activation of other inflammatory signalling pathways via MAPK and activation of NF-kB itself^1,2^. TNFα signalling is mediated through binding to either TNFRSF1A (Tnfr1) or TNFRSF1B (Tnfr2). Both TNF receptors can activate MAPK and NF-kB, although with different dynamics and intensity, with canonical signalling via TNFRSF1A dominant for most cell types^3^.

Polarisation of immune cells occurs to either promote or inhibit inflammation. NF-kB is generally considered to promote a pro-inflammatory phenotype in macrophages and drive expression of TNFα in mice and humans^4–6^. A key element of models for NF-kB signalling is IKK2 phosphorylation, resulting in IkBalpha (IkBA) degradation and release of NF-kB heterodimers, which can then translocate into the nucleus^7^. An inhibitor of IKK2, BMS-345541, inhibits release of TNFα, IL1beta, IL6 and IL8 in the human monocyte cell line THP-1^8^. Induced expression of a super-repressor version of IkBA (IkBA-SR) likewise represses inflammatory cytokine production and prevents p65 nuclear localisation^9^, but does not inhibit expression of anti-inflammatory cytokines IL-10 or IL-11^10^. This implies that NF-kB is required for pro-inflammatory cytokine expression, but is not required for expression of anti-inflammatory cytokines.

Macrophage migration is an important requirement for their function as part of the immune response to both infection and injury. Damage and infection-induced signals, such as redox oxygen species, changes to osmolality or release of HMGB1 can activate inflammatory cells, which then respond by directional migration^11–15^.

NF-kB is an important regulator of many genes with roles in cell migration, including matrix remodelling enzymes MMP9 and MMP13 expressed in macrophages^16,17^. In vitro studies reveal that canonical NF-kB activity is important for directional migration of macrophages towards a source of CXCL12, but not towards TNFα^18,19^.

Cytokines such as TNFα and IL1 have well-described roles in inducing pro-inflammatory profiles in macrophages through activation of TLR signalling. There is less known about their potential roles in regulating immune cell migration, however. The *Drosophila* TNFα orthologue *Eiger* regulates macrophage migration through modifying myosin in ectodermal cells^20^. In mammals, TNFα can modify integrin function and actin bundling in fibroblasts, and promote myoblast migration in vivo via up-regulation of MMP9^21–23^.

Injury in zebrafish larvae results in up-regulation of a TNFα reporter in macrophages migrating to the injury^24,25^. Expression of *tnfa* in macrophages has been used as an indicator of pro-inflammatory polarity and is associated with rapid migration and stellate morphology^24–27^. This association between TNFα expression and macrophage dynamics suggests that the activity of inflammatory pathways may be coupled with their migratory behaviour. Given that NF-kB can regulate cell migration in vitro, we hypothesised that a feedback loop between TNFα and NF-kB may coordinate macrophage migration and inflammation. We have investigated this using the paradigm of immune cell recruitment in response to tissue injury in the caudal tailfin of zebrafish larvae. Using manipulation of gene function and signalling activity, we demonstrate that NF-kB acts to limit TNFα activity in macrophages responding to tailfin injury. Inhibition of NF-kB results in an elevated expression of TNFα and a more directional migration of macrophages to injured tissue. This is driven by elevated TNFα activity and can be rescued by inhibition of TNFα signalling. A consequence of deregulated TNFα activity is a prolonged inflammatory environment at the wound blastema and impaired regeneration. Our results suggest that NF-kB acts as a brake to uncontrolled TNFα signalling in an autocrine manner within macrophages, thus controlling the perdurance and extent of pro-inflammatory signalling during regeneration.

## Results

### NF-kB signalling shows a spatiotemporal activation in inflammatory cells relative to the injury

**Fig. 1:**
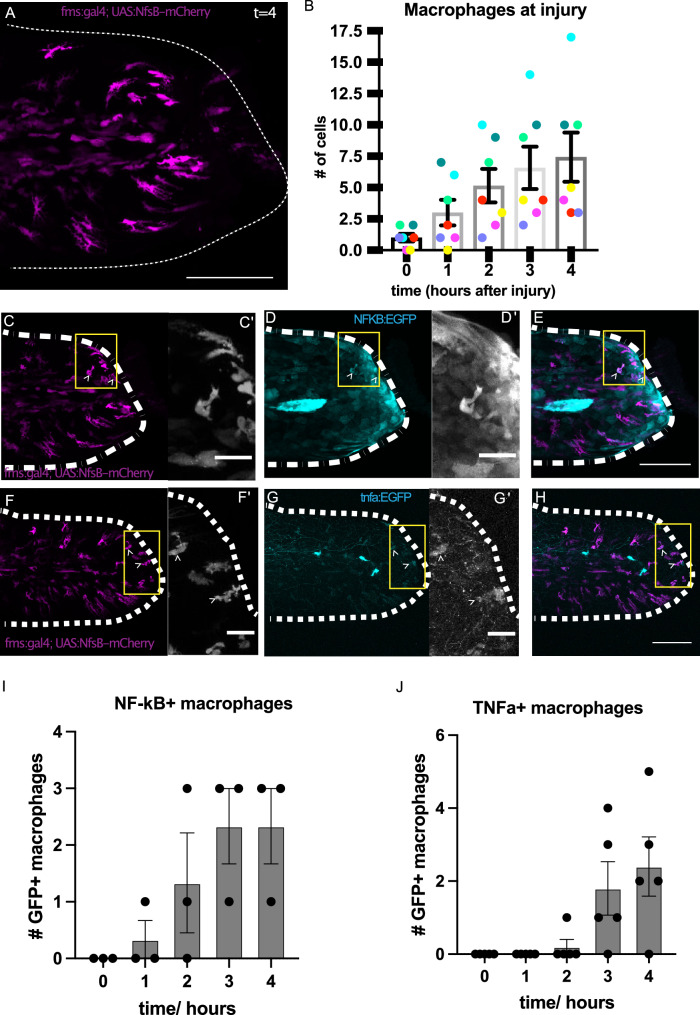
Tail fin transection induces a temporal-spatial activation of NF-kB and TNFα in macrophages migrating to the injury. Macrophages in transgenic larvae expressing fms:gal4; UAS:NfsB-mCherry were tracked at the site of tailfin amputation over time (*t*) in hours post-amputation (hpa) and number of macrophages present at the injury plotted (**A**, **B**, *n* = 7 animals, colours represent individual animals). Macrophages expressing NFKB:EGFP were visualised and quantified at 1, 2, 3, 4 hours post-amputation (**C**–**E**, **I**, *n* = 3 animals). Macrophages expressing tnfa:egfp were visualised and quantified at 1, 2, 3 and 4 hours post-amputation (**F**–**H**, **J**, *n* = 6 animals). Bar plots show mean values with bars representing the standard error of the mean. Scale bars 100 µm (**A**, **C**, **D**, **E**, **F**, **G**, **H**) or 25 µm (**C’**, **D’**, **F’**, **G’**).

In order to determine where and when NF-kB could act to regulate inflammatory cell behaviour in response to tissue injury, we obtained a spatiotemporal profile of macrophages responding to a tailfin amputation at 3 days post-fertilisation (dpf) (Fig. 1A, Supplementary Table 1). In our injury model at 4 hours post-amputation (hpa) there are on average 7.4 ± 1.9 macrophages at the injury site (Fig. 1B).

Reporter lines for NF-kB activity (NFKB:EGFP) and *tnfa* expression (tnfa:egfp) were used to map activity of pro-inflammatory signalling relative to the injury. Following tail amputation, macrophages recruited to injured tails showed NFKB:EGFP expression from 2 hpa (Fig. 1C–E, I, Supplementary Movie S1, Supplementary Table 2). Activity of the tnfa:egfp reporter was similarly active from 2 hpa in macrophages arriving at the injured tail (Fig. 1F–H, J, Supplementary Movie S2, Supplementary Table 3). This reveals that NF-kB activity and *tnfa* expression are initiated in macrophages in a temporal manner as they respond and are recruited to the injury. We confirmed this by showing that macrophages at the injury site showed NF-kB activity using an antibody to p65, which revealed nuclear localisation in cells (Supplementary Fig. 1).

### Small molecule inhibitors of IKK2 and p65 repress canonical NF-kB signalling

**Fig. 2:**
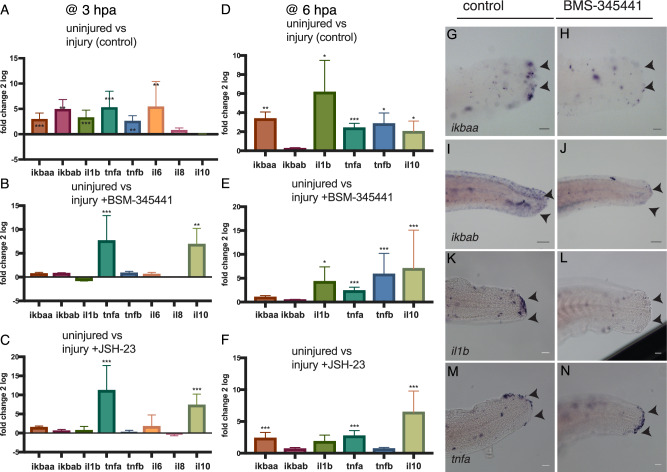
Treatment with NF-kB inhibitors results in an abnormal inflammatory gene expression profile in the regenerating fin. Uninjured or tail amputated 3 dpf larvae were treated with DMSO (control), 2.5 mM BMS-345541 or 300 µM JSH-23 until 3 hpa (**A**–**C**) or 6 hpa (**D**–**F**). Extracted RNA from pooled animals (*n* = 60 animals/condition) was used as template for RT-qPCR and fold difference plotted for injured relative to uninjured animals for each condition. Gene expression of *ikbaa* (**G**, **H**), *ikbab* (**I**, **J**), il1b (**K**, **L**) and *tnfa* (**M**, **N**) was examined by in situ hybridisation in control (**G**, **I**, **K**, **M**) and BMS-354451 treated (**H**, **J**, **L**, **N**) larvae at 3 hpa; arrows indicate expression at the wound site. Significance for relative gene expression difference between conditions was calculated using the REST software after correcting for primer efficiency (**A**–**F**). Significance for differences between groups is calculated using a randomisation test in REST, which compares across conditions (***p < 0.001, **p < 0.01, *p < 0.05, n.s. not significant). Bar plots show mean values with bars representing the standard error of the mean. Scale bars 100 µm (**G**–**N**).

We aimed to test the importance of NF-kB activity in controlling immune responses to injury. A baseline response to injury was obtained by measuring gene expression at the amputated tail relative to non-amputated tails using quantitative RT-PCR. Expression of *il1b*, *il6, tnfa, tnfb* and IkBalpha orthologues *nfkbiaa* (*ikbaa*) and *nfkbiab (ikbab)* was up-regulated at the amputation site at 3 hpa, but *il8* did not show elevated expression (Fig. 2A). At a later stage (6 hpa) *ikbaa*, *il1b*, *tnfa*, *tnfb* and *il10* are up-regulated, but neither *il6* or *il8* were detectable (Fig. 2D). To temporally manipulate the pathway we assessed how two previously described inhibitors of NF-kB signalling affected gene expression and NF-kB activity in uninjured larval tails. BMS-345541 is a specific inhibitor of IKK2 and, to a lesser extent, IKK1^8^; JSH-23 inhibits p65 nuclear translocation^28^. Both inhibitors have been shown to effectively abrogate NF-kB activity in zebrafish^29,30^.

Treatment of larvae with BMS-345541 at 2.5 mM or JSH-23 at 300 µM resulted in reduced expression of NF-kB target genes *il1b*, *tnfa* and *ikbaa* and *ikbab* in the tail in the absence of injury relative to control animals at 3 hpa (Supplementary Fig. 2, Supplementary Table 4). In injured animals treated with BMS-345541 or JSH-23, expression of *il1b*, *il6, ikbaa* and *ikbab* in the tail is all reduced relative to control animals at 3 hpa (Fig. 2A–C). In contrast *tnfa* expression is up-regulated relative to non-treated control animals. We also observe increased expression of the anti-inflammatory cytokine *il10* in inhibitor-treated animals. At 6 hpa, *tnfa*, *il10* and *tnfb* expression is elevated in BMS-345541-treated animals relative to control animals, reflecting a prolonged inflammatory environment in response to inhibition of NF-kB (Fig. 2D, E). Animals treated with JSH-23 showed a similar up-regulation of *tnfa* and *il10* at 6 hpa, but did not show up-regulation of *tnfb* (Fig. 2F).

To determine where the changes to gene expression identified by qPCR occur in the injured tail injury we examined gene expression by in situ hybridisation. In the presence of BMS-345541, there is no expression of *ikbaa* or *ikbab*, nor *il1b* following fin amputation, in agreement with results from qRT-PCR analyses (Fig. 2G–L). In contrast, *tnfa* is still expressed throughout the wound blastema (Fig. 2M, N). These results reveal that inhibition of NF-kB activity results in elevated *tnfa* expression in the context of injury.

**Fig. 3:**
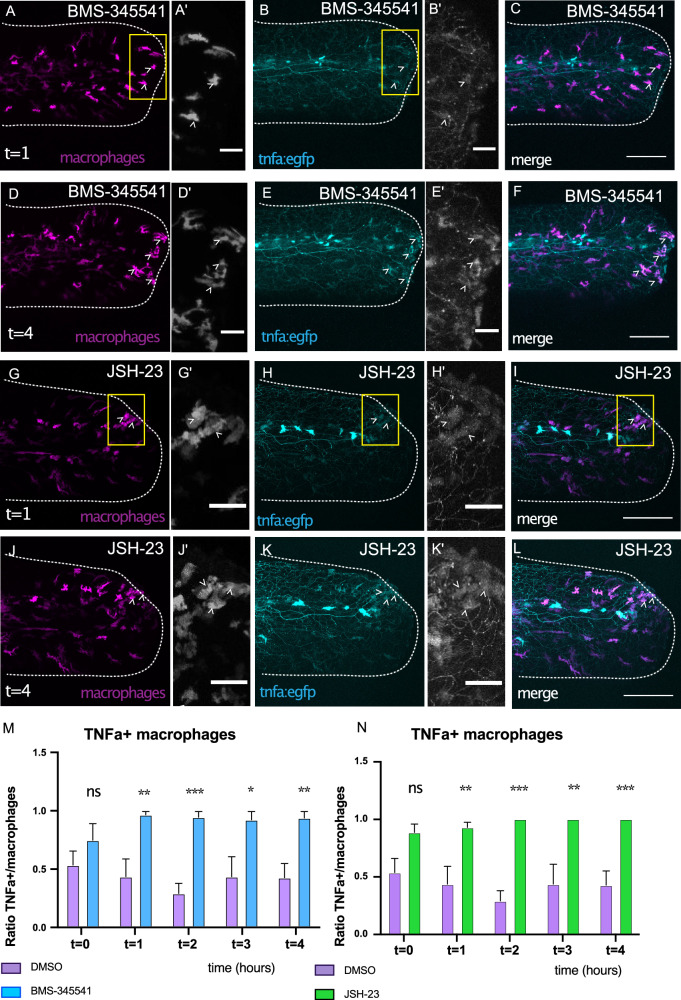
*TNFα* expression is elevated in macrophages responding to injury when NF-kB signalling is inhibited. Tailfin injuries were performed on transgenic larvae expressing tnfa:egfp reporter and animals exposed to 2.5 mM BMS-345541 (**A**–**F**) or 300 µM JSH-23 (**G**–**L**). Animals were observed by time-lapsed microscopy and macrophages (magenta) expressing tnfa:egfp (cyan) in the tailfin (magnified region) visualised at 1 or 4 hpa, transformed to positive values and expressed as a ratio. The difference in proportion of macrophages expressing tnfa:egfp reporter between conditions was tested by 1-way ANOVA with Dunnett’s multiple comparison test at each time point (**M**, **N**, *n* = 4-6 animals per condition). Significance for differences between treated and control samples is shown (***p < 0.005, **p < 0.01, *p < 0.05, n.s. not significant). Bar plots show mean values with bars representing the standard error of the mean. Scale bars 100 µm (**A**–**L**) or 25 µm (**A’**, **B’**, **D’**, **E’**, **G’**, **H’**, **J’**, **K’**).

To determine in which cells elevated *tnfa* expression occurred in response to inhibition of NF-kB signalling, we examined the expression of the NFKB:EGFP and tnfa:egfp reporter lines in macrophages labelled by expression of fms:gal4; UAS:NfsB-mCherry. We find that in the presence of BMS-345541 and JSH-23, there was an increased number of macrophages that express the tnfa:egfp reporter at the injured tail (Fig. 3, Supplementary Movies S3 and S4, Supplementary Table 5). In contrast, the NFKB:EGFP reporter is never expressed at the injury site when animals were exposed to either compound (Supplementary Fig. 3).

### IKK2 and p65 regulate macrophage migration

**Fig. 4:**
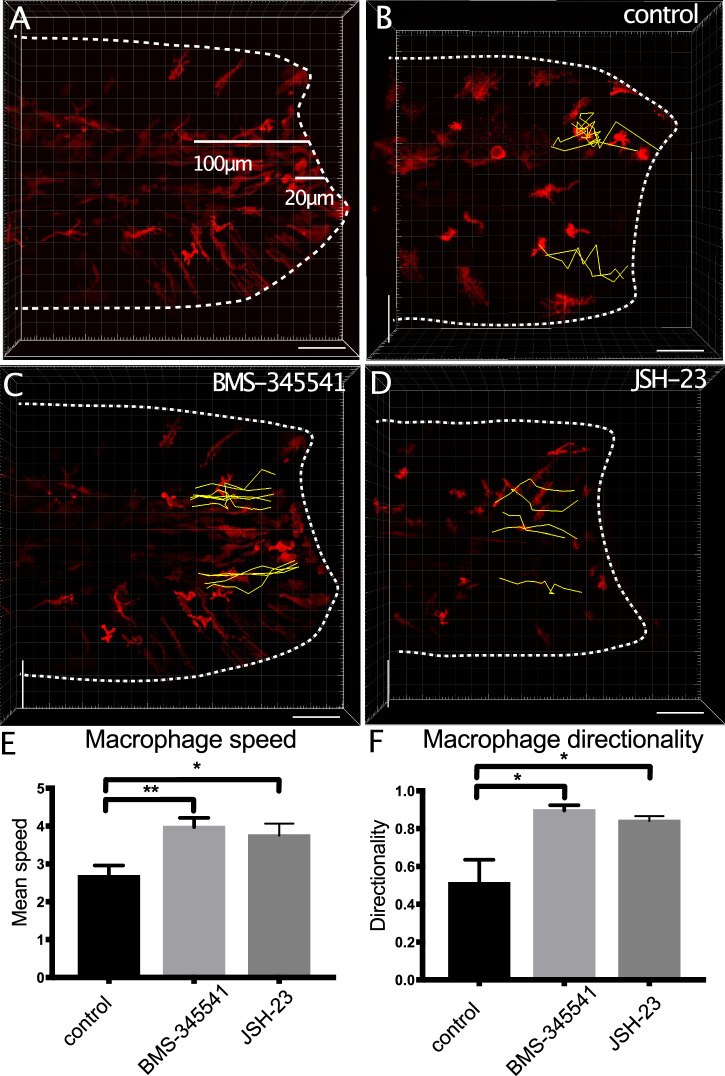
Macrophage responses to injury are altered in the presence of IKK inhibitors. Macrophages within 100 µm of the injury were observed by time-lapsed microscopy for 4 hpa after tailfin injury of 3 dpf larvae (**A**). Macrophage migration was tracked towards the fin fold (outlined) using Imaris (yellow lines) to generate measures of instantaneous speed and directionality (**B**–**D**). Injured 3 dpf larvae expressing fms:gal4; UAS:NfsB-mCherry (red) were treated with DMSO (control, **B**), 2.5 mM BMS-345541 (**C**) or 300 µM JSH-23 (**D**) immediately after amputation. Speed (**E**) or directionality (**F**) of each cell in animals for each condition was plotted, and the significance of differences between control and drug-treated animals was tested by 1-way ANOVA with Dunnett’s test (*n* = 6 animals/condition). Significance is shown (***p < 0.005, **p < 0.01, *p < 0.05, n.s. not significant). Bar plots show mean values with bars representing the standard error of the mean. Scale bar 100 µm (**A**–**D**).

The increased expression of *tnfa* in macrophages of animals treated with NF-kB inhibitors suggests these cells have a different inflammatory profile. To test whether this affected macrophage responses to injury, we tracked macrophages migrating to the tail (100 µm from the wound blastema edge) and quantified cell movement (Fig. 4A). In control animals, macrophages display a wandering or meandering movement as they migrate towards the injury (Fig. 4B, Supplementary Movie S5). In contrast, in BMS-345541 or JSH-23-treated larvae, macrophages showed a more directional movement (Fig. 4C, D, Supplementary Movies S6 and S7). Speed and directionality were increased and cells arrived at the injury at an earlier time after injury than in control larvae (Fig. 4E, F, Supplementary Table 6). This altered behaviour relative to inhibition of NF-kB was only apparent in the context of an injury, as in uninjured tissues, the behaviour of patrolling macrophages was unaffected (Supplementary Fig. 4, Supplementary Movies S8–S10).

### TNF receptor 1A is required for exploratory macrophage behaviour

The NF-kB pathway is a major target of TNFα signalling and this is primarily mediated through TNF receptor 1 (TNFRSF1A) in mammals. To determine whether TNFα signalling is important for mediating NF-kB activity and so controlling macrophage cell behaviour, we identified a zebrafish mutant for TNF receptor 1A (*tnfrsf1a*) from an ENU mutagenesis screen. Zebrafish *tnfrsf1a*^*sa8496*^ mutants have a G > T transversion at the splice acceptor site of exon 3 which is predicted to cause aberrant splicing to a cryptic splice site in exon 4 (Supplementary Fig. 5). Translated proteins will contain premature stop codon due to a frameshift in the open reading frame resulting a predicted protein lacking the majority of the Cysteine rich or death-like domains. Homozygous mutants express altered *tnfrsf1a* transcripts but do not show any overt phenotypes and survive up to 12 dpf without any increased mortality compared to WT siblings (Supplementary Fig. 6).

**Fig. 5:**
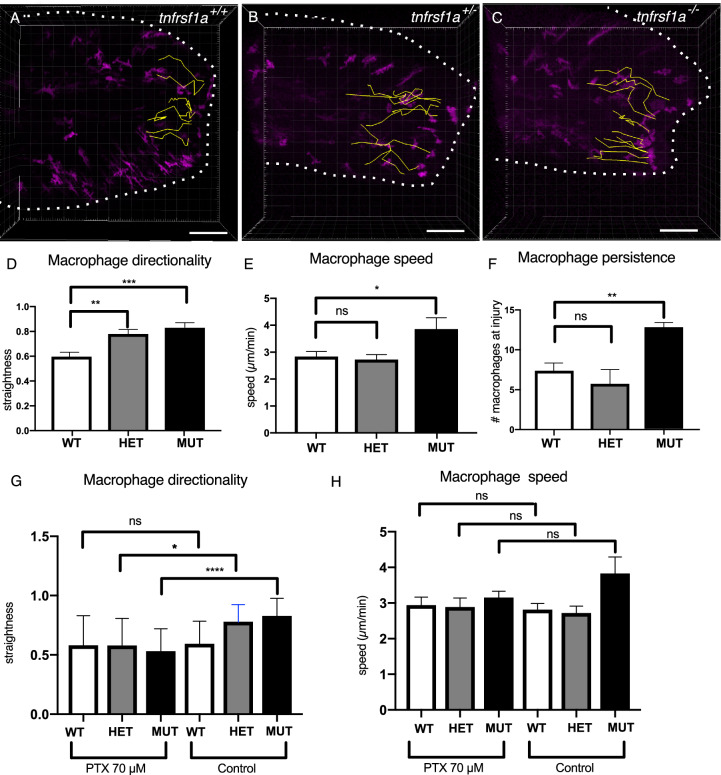
Macrophage responses to injury are altered in *tnfrsf1a*^*sa8496*^ mutants. Macrophage responses in larvae with varying *tnfrsf1a* genotypes were visualised by time-lapsed microscopy for 4 hpa (**A**–**C**). Cell behaviour was quantified using Imaris (yellow lines) and measures of cell directionality (**D**), instantaneous speed (**E**), and persistence (**F**) obtained for mutant (MUT), heterozygotes (HET) and wild-type animals (WT). Macrophage responses to injury were examined in larvae with varying genotypes in the presence of 70 µM PTX or in untreated control larvae and directionality (**G**) and speed (**H**) were quantified. Significance of differences between genotypes for directionality and speed was tested by a two-tailed unpaired Student’s *t*-test (*n* = 3 animals/genotype). Significance is shown (***p < 0.005, **p < 0.01, *p < 0.05, n.s. not significant). Bar plots show mean values with bars representing the standard error of the mean. Scale bar 100 µm (**A**–**C**).

We tracked macrophages responding to tail amputation in *tnfrsf1a* mutants and siblings as previously and noted a similar phenotype as in animals treated with NF-kB inhibitors: a more rapid and persistent migration to the injury (Fig. 5A–C, Supplementary Movies S11–S13). This was significantly different between mutant, heterozygote and wild-type animals, reflecting an allele-dosage-dependent effect (Fig. 5D–F). This difference in macrophage behaviour relative to *tnfrsf1a* gene dosage may reflect the relative level of TNFα signalling occurring in response to injury. We therefore tested whether abrogation of TNFα signalling altered macrophage responses in animals with differing dosages of *tnfrsf1a* gene function. To inhibit TNF signalling, we used the small molecule Pentoxifylline (PTX), previously shown to inhibit *tnfa* transcription in zebrafish^31^. Larvae with various *tnfrsf1a* genotypes were injured and treated with PTX or were untreated (control) and macrophages were imaged. We observed in wild-type animals that there was no difference in macrophage behaviour compared to untreated animals (Fig. 5G, H). In contrast, macrophage behaviour was significantly different between *tnfrsf1a* mutant larvae treated with PTX compared to untreated mutants. Cell speed and directionality were indistinguishable between mutants and wild-type animals in the presence of PTX, revealing that inhibition of TNFα signalling was able to rescue normal macrophage behaviour in the absence of tnfrsf1a function (Supplementary Table 7).

### NF-kB acts autonomously to regulate macrophage migratory behaviour

**Fig. 6:**
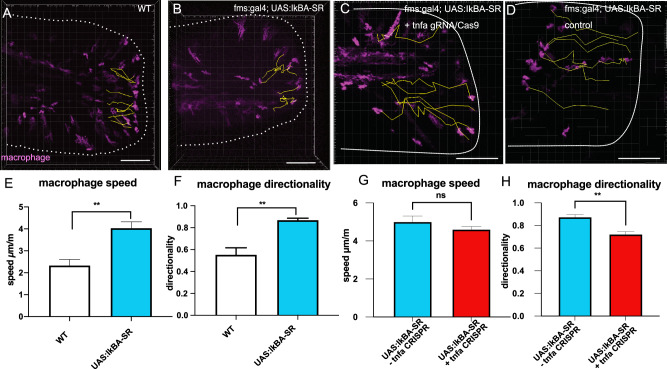
Macrophage-specific inhibition of NF-kB activity perturbs migration in a TNFa-dependent manner. Macrophage responses in larvae expressing IkBA-SR in macrophages (**B**) and control animals without the transgene (**A**, WT) were visualised by time-lapsed microscopy for 4 hpa. Macrophages were visualised in larvae expressing IkBA-SR in macrophages and injected by Cas9 and gRNA to *tnfa* (**C**, +tnfa CRISPR) or in sibling uninjected control larvae (**D**). Cell behaviour relative to the injury (white line) was quantified using Imaris (yellow lines) and measures of instantaneous speed (**E**, **G**), and directionality (**F**, **H**) compared between conditions. Significance of difference between conditions for speed and directionality was tested by two-tailed unpaired Student’s *t*-test (WT vs fms:gal4; UAS:IkBA-SR *n* = 3 animals/condition, fms:gal4; UAS:IkBA-SR vs fms:gal4; UAS:IkBA-SR + tnfa gRNA/Cas9 *n* = 6 animals/condition). Significance is shown (***p < 0.005, **p < 0.01, *p < 0.05, n.s. not significant). Bar plots show mean values with bars representing the standard error of the mean. Scale bar 100 µm (**A**–**D**).

**Fig. 7:**
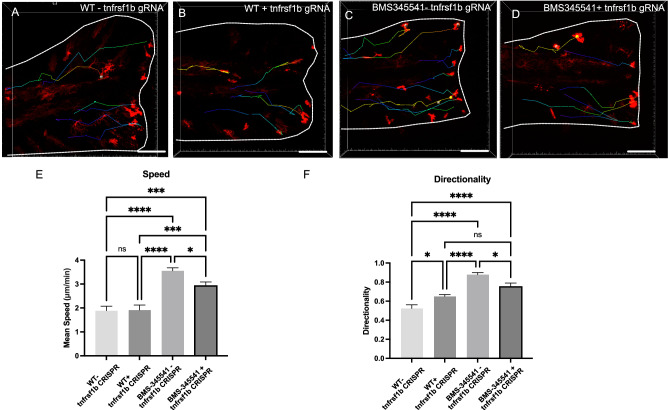
Mutagenesis of *tnfrsf1b* reduces the effects of NF-kB inhibition on macrophage migration. Macrophage responses in control animals (**A**, WT) and in animals injected by Cas9 protein and gRNAs to *tnfrsf1b* (**B**) were visualised by time-lapsed microscopy for 4 hpa. Macrophages were visualised in uninjected larvae exposed to BMS-345541 (**C**) and in those injected by Cas9 and gRNA to *tnfrsf1b* (**D**, +tnfa CRISPR). Cell behaviour relative to the injury (white line) was quantified using Imaris (coloured lines) and measures of instantaneous speed (**E**), and directionality (**F**) compared between conditions. Significance of difference between conditions for speed and directionality was tested by 2-way ANOVA using Tukey’s test for multiple comparisons (*n* = 3 animals/ condition). Significance is shown (***p < 0.005, **p < 0.01, *p < 0.05, n.s. not significant). Bar plots show mean values with bars representing the standard error of the mean. Scale bar 100 µm (**A**–**D**).

The similar macrophage behaviours elicited by abrogation of NF-kB activity and *tnfrsf1a* suggested that NF-kB may act to regulate normal macrophage responses to injury in a TNFa-dependent manner. In order to determine whether NF-kB acts autonomously to regulate macrophage responses to injury, we generated a transgenic line in which a dominant active version of IkBalpha (IkBA-SR) is expressed under the control of a UAS promoter (Supplementary Fig. 7). In mammals, super-repressor versions of IkBA are resistant to ubiquitination and so act to prevent dissociation of p65 from the inhibitor complex, including IKK, thereby inhibiting p65 nuclear translocation^9^. We over-expressed IkBA-SR in macrophages and measured their response to tail amputation. Similar to animals treated with NF-kB small molecule inhibitors, or *tnfrsf1a* mutants, we noted that macrophages showed a more directional and rapid migration to the injury (Fig. 6A, B, E, F, Supplementary Movies S14 and S15). To test whether elevated *tnfa* expression is important for the increased migration and persistence of macrophages when NF-kB activity was lost, we evaluated the consequences of loss of TNFα function using PTX. We found that PTX treatment reduced macrophage speed and directionality in animals over-expressing IkBA-SR in macrophages and control animals (Supplementary Fig. 8, Supplementary Table 8). To confirm this was caused by loss of TNFα signalling, we mutated *tnfa* by CRISPR/Cas9 (Supplementary Fig. 9). Mutagenesis of *tnfa* did not affect macrophage speed or directionality in wild-type animals (Supplementary Fig. 10, Supplementary Table 9). In larvae over-expressing IkBA-SR in macrophage cells, directionality, but not speed, was reduced by CRISPR/Cas9 mutagenesis of *tnfa* (Fig. 6C, D, G, H, Supplementary Movies S16 and S17). Given that NF-kB-dependent changes to macrophage migration are related to elevated TNFα signalling, but loss of tnfrsf1a function causes a similar phenotype as for loss of NF-kB activity, we wondered whether this was due to TNFα signalling through TNF receptor 1B. To test this, we mutagenesised *tnfrsf1b* using gRNAs designed to exon 1 (Supplementary Fig. 11) in the context of NF-kB inhibition, using BMS-345541, and examined the effects on macrophage responses to tailfin amputation. In control animals, mutagenesis of *tnrsf1b* did not affect macrophage migration (Fig. 7A, B, E, F, Supplementary Movies S17 and S18). In contrast, macrophage directionality and speed were reduced in BMS-345541-treated animals injected by gRNA to mutagenesise *tnrsf1b* compared to uninjected treated animals (Fig. 7C, D, E, F, Supplementary Movies S19 and S20, Supplementary Table 10). This reveals that altered macrophage dynamics in the context of NF-kB inhibition are dependent on tnf receptor 1b signalling.

### Inhibition of NF-kB signalling in macrophages retards fin regeneration

**Fig. 8:**
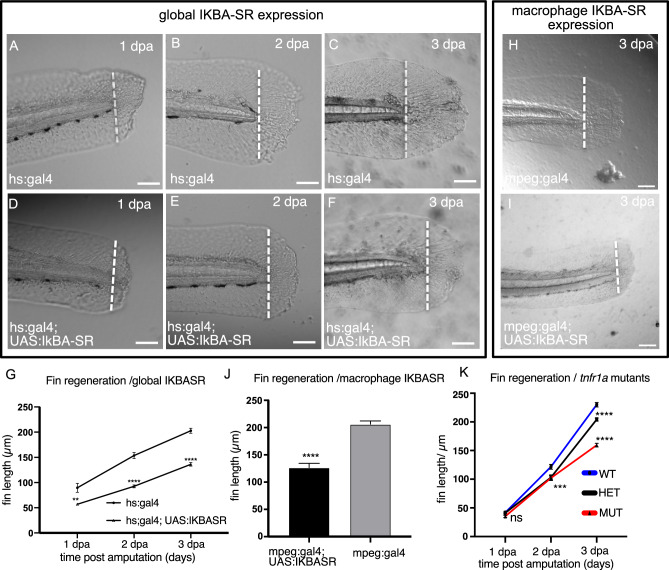
Tailfin regeneration is impaired when NF-kB activity is inhibited through IkBA-SR expression. Regrowth of the fin fold in 4 dpf larvae was measured relative to the notochord (dotted line) at 1 (**A**, **D**), 2, (**B**, **E**), 3 (**C**, **F**) days post-amputation (dpa) in control (**A**–**C**) and transgenic animals expressing IkBA-SR globally through induction of a heat-shock promoter (hs:gal4) from 0 dpa (**D**–**F**). Fin regrowth was likewise measured in control (**H**) and transgenic larvae expressing IkBA-SR in macrophages (**I**) at 3 dpf. A plot of fin length relative to the notochord at 1, 2, 3 dpa reveals a significantly reduced regrowth of the fin in larvae expressing IkBA-SR (**G**, *n* = 22 and 28 for each genotype). Fin regrowth at 3 dpa was reduced in larvae expressing IkBA-SR in macrophages relative to control animals (**J**, *n* = 22 and 21 for each genotype). Plots of fin growth in wildtype (*tnfrsf1a*+*/+*), heterozygote (*tnfrsf1a*+*/−*) and mutant larvae (*tnfrsf1a−/−*) at 1, 2 and 3 dpa reveal an allele-dependent regeneration (**K**). Statistical comparisons were performed by 2-way ANOVA with Sidak’s multiple comparison to identify the significance of difference across conditions. Significance is shown (***p < 0.005, **p < 0.01, *p < 0.05, n.s. not significant). Bar plots show mean values with bars representing the standard error of the mean. Scale bars 100 µm (**A**–**F**, **H**, **I**).

Regulation of NF-kB signalling is important for effective regeneration of the adult heart and fins^32,33^. To understand the importance of NF-kB for regeneration, we measured fin regrowth relative to the notochord at 1, 2 and 3 days after injury in animals expressing the IkBA-SR globally in a temporally controlled manner using a heat-shock promoter (hs:gal4). Expression was induced by heat shock prior to injury (*t* = 0) and every 24 h thereafter until 3 days after injury (Fig. 8A–F). We found that in animals expressing IkBA-SR, regeneration was impaired relative to non-transgenic control animals (Fig. 8G). We then tested whether macrophage-specific inhibition of NF-kB activity would alter fin regeneration by expressing IkBA-SR in macrophages (Fig. 8H, I). Similar to animals with a global repression of NF-kB, there was delayed fin growth in animals in which IkBA-SR is specifically expressed in macrophages (Fig. 8J). To understand whether TNF receptor 1A function is required for fin regeneration, we measured fin growth after amputation in larvae from *tnfrsf1a* heterozygote incrosses. We found that *tnfrsf1a* mutants show reduced growth compared to heterozygotes or wild-type animals, similar to animals over-expressing IkBA-SR (Fig. 8K, Supplementary Fig. 12).

To understand why regeneration is delayed, we examined proliferation of cells at the wound blastema at 1, 3 or 6 h after injury when animals were treated with NF-kB inhibitors. In uninjured animals, there was no difference in the relative proportion of BrdU incorporation in the fin of animals exposed to NF-kB inhibitors compared to control animals. In contrast, in the context of injury, we observed elevated proliferation in animals exposed to NF-kB inhibitors relative to injured control animals at 3 hpa (Supplementary Fig. 13). At 6 hpa, proliferation was higher in BMS-345541-treated larvae, but not JSH-23-treated animals. In contrast, *tnfrsf1a* mutants showed a lower proliferative response relative to wild-type and heterozygous animals (Supplementary Fig. 13).

## Discussion

**Fig. 9:**
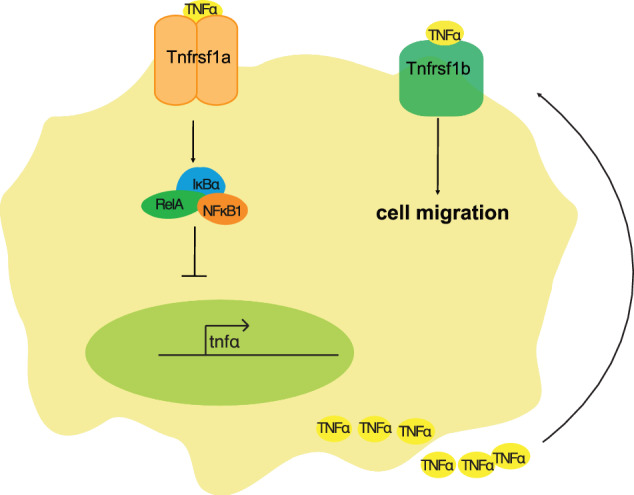
A model for the relationship between NF-kB signalling and macrophage migration in the context of tissue damage. Signalling by TNFα through TNF receptor 1a (tnfrsf1a) activates NF-kB, which represses *tnfa* expression in macrophages. A loss of NF-kB activity or of tnfrsf1a results in elevated expression of *tnfa*, resulting in elevated TNFα release and activation of TNF receptor 1b (tnfrsf1b). This results in increased motility of macrophages responding to tissue injury.

NF-kB is a master regulator of inflammation and has been targeted for treating diseases involving chronic inflammation or aberrant immune cell function. We show that reduced activity of NF-kB alters macrophage responses to injury in a TNFa-dependent manner. This results in an amplification of a feed-forward loop and perturbs the inflammatory environment at the wound blastema, resulting in impaired regeneration in conjunction with elevated proliferation of wound blastema cells. Inhibition of the NF-kB to TNFα negative feedback loop results in an exaggerated inflammatory response that impairs tissue repair. We also find an unexpected relationship between NF-kB and TNFα activity in regulating cell migration, with elevated TNFα causing a more directional movement of macrophages (Fig. 9). To our knowledge, this is the first evidence of TNFα acting to directly regulate cell behaviour in vivo, with implications for a number of biological contexts in which TNFα expression is elevated, including several chronic diseases, cancers and ageing.

As a master regulator of inflammation, it is well known that many pro-inflammatory genes have kB binding motifs in their promoters that enable control of expression by various NF-kB dimers^34^. TNFα is a transcriptional target of NF-kB and thus used as a readout of NF-kB activity in many cells^35,36^. A number of chronic diseases and conditions involving elevated TNFα activity reveal that although TNFα induction by NF-kB is a standard part of the inflammatory response to injury or activity, expression needs to be modulated to prevent tissue damage or death^37^.

Knockout of IKK2 in macrophages in a mouse model of atherosclerosis revealed a reduction of TNFα expression and strong loss of IL-10 in macrophages when challenged by LPS^38^. Likewise, we observe inhibition of NF-kB in uninjured animals represses *tnfa* and *il10* expression in macrophages of fish. In contrast, there is an up-regulation of *tnfa* and *il10* expression in macrophages when NF-kB is inhibited by IKK2 inhibitors. The key difference in these experiments is that we assayed the effects of NF-kB inhibition in macrophages responding to a directional damage signal, rather than non-migratory cells in vitro. This suggests that tissue damage dramatically alters how NF-kB regulates these two important cytokines during responses to tissue injury.

Mathematical models for NF-kB and TNFα co-regulation use the logic that these two pathways self-regulate each other’s activity via positive and negative feedback loops. Specifically, increased TNFα release will induce increased NF-kB activity, which is in turn regulated by inhibitors A20 and IkBa, which are induced by NF-kB^39,40^. TNFα expression is modulated by a number of different signalling pathways, including MAPK, which activates AP-1^41,42^. Macrophages exposed to bacterial proteins show dose-dependent responses of the NF-kB and MAPK pathways that result in an expression switch mediated by MAPK signalling above a certain threshold^43^. Even when NF-kB activity is induced using a constitutively active IKK2, MAPK activation of p38 is required for increased TNF expression^44^. TNFα expression is increased in response to MAPK signalling and can induce NF-kB in neighbouring cells. It is possible, therefore, that the elevated TNFα expression we observed in NF-kB deficient cells is due to elevated MAPK signalling. Intriguingly, modelling and testing the response of fibroblasts to sequential inflammatory cytokines reveals that an NF-kB-mediated feedback loop can repress Myd88-mediated signalling^45^. If NF-kB activity is inhibited, but other inflammatory cytokines are able to activate Myd88 and downstream signalling, including MAPK, this implies NF-kB is acting to repress *TNFα* expression beyond a certain threshold.

Our results reveal an unexpected role for NF-kB in regulating *tnfa* expression in macrophages when responding to tissue injury. We do not see expression of *tnfa* in macrophages of uninjured animals and this is not changed by the addition of NF-kB inhibitors. Given that NF-kB is traditionally thought to drive expression of TNFα in a feedback loop, we had hypothesised that an inhibition of NF-kB would result in diminished NF-kB activity and hence TNFα expression. In contrast, we found that inhibition of NF-kB results in elevated *tnfa* expression in macrophages in an injury context. This implies that NF-kB acts to modulate TNFα activity in macrophages responding to injury through a feedback regulatory loop. The membrane-bound version of TNF (mTNF) is cleaved by TACE to release secreted TNF (sTNF), which is thought to promote the paracrine activity in other cells^46,47^. Activity of membrane-bound TNFα is associated with activation of both TNFRSF1A and TNFRSF1B^48^. Our results cannot resolve whether TNFα is acting in an autocrine or paracrine manner in macrophages to drive the altered behaviours observed in response to NF-kB inhibition. Given that we observe similar behaviour in the *tnfrsf1a* mutant, this suggests that altered macrophage behaviour is driven by autocrine signalling through tnfrsf1b.

Models for TNFa–NF-kB activity propose feedback loops between these pathways to regulate their respective activity. Our results show that these can be decoupled with elevated TNFα associated with an altered immune response to injury. The increased speed of migrating macrophages in response to elevated TNFα signalling may be explained through a number of mechanisms, including changes to interactions with the local environment, altered actinomyosin dynamics or altered responses to secreted factors. TNFα is required for FAK activity and induction of IL-6 expression in a variety of cell types^49^ and promotes migration of synovial fibroblasts via FAK^21^. This appears to be mediated by integrin signalling through activity of cholesterol 25-hydoxylase, an enzyme that generates 25-hydoxycholesterol, which acts as a ligand for integrins and induces FAK activity^22^. Intriguingly, inhibiting Integrin alpha5 beta1 activity in macrophages resulted in reduced NF-kB activity in response to TNFα, implying that signalling via integrins to FAK dictates how immune cells respond to TNFα activity^22^. Cell geometry is an important determinant for how cells respond to TNFα. In a series of elegant experiments Mitra et al. showed that fibroblasts adhering to different-shaped micropatterned substrates showed differential responses to TNFα^50^. Cells not under tension (circular) showed a faster response to TNFα than stretched cells, with a correspondingly greater nuclear translocation of p65. TNFα also induced F-actin depolymerisation and this was stronger in stretched cells with a corresponding decrease in Rho activity. Tension due to F-actin polymerisation, therefore, appears to be an important determinant for how cells respond to TNFα. Macrophages employ a mesenchymal mode of migration in response to injury, dependent on RhoA signalling and mediated by adhesions^51^. Actin turnover and myosin contractility are key determinants of mesenchymal cell migration, with adhesion to the extracellular matrix acting as a brake on migration speed. In larvae mutant for L-Plastin, macrophages responding to tissue injury show a slower migratory response, potentially due to impaired integrin turnover resulting in greater adhesion^52^. Given that TNFα is able to modulate both integrin signalling and F-actin polymerisation, it is possible that the more rapid migratory behaviour we observe in animals with elevated TNFα expression arise due to more rapid cycling of actin and altered adhesion dynamics. Another factor governing macrophage migration is activity of matrix metalloproteases (MMPs) that degrade the extracellular matrix to enable passage. Inhibition of MMPs increased speed and directionality of macrophages, suggesting that altered interactions with the ECM change the mode of cell migration^53^. A number of MMPs are transcriptionally regulated by NF-kB, including MMP1, MMP9, MMP13^16,17^.

Elevated and prolonged TNFα activity at the wound blastema is refractory to regeneration and resolution of inflammation^54^. This suggests that feedback loops exist to temporally limit when and where TNFα activity occurs during regeneration. A transgenic reporter line for *tnfa* expression (tnfa:egfp) was used to show that immune cells recruited to the injured caudal tailfin show TNFα activity^25^. Quantification of the macrophage population at the injury of tnfa:egfp transgenic larvae revealed a temporal shift from pro-inflammatory (tnfa:egfp) to anti-inflammatory macrophages. To test whether the dynamics of immune cell responses to injury affect tissue regeneration, Miscolci et al. applied thermal insult to the tail of tnfa:egfp larvae to induce extensive damage. They observed a prolonged retention of tnfa:egfp+ macrophages at the injury commensurate with a failure to regenerate the tail^54^. This corresponds with our observations of tail regeneration in animals in which NF-kB has been inhibited, which show an increased expression of *tnfa* in macrophages. We were able to show that impaired regeneration of the fin was a direct consequence of NF-kB inhibition in macrophages by overexpression of a constitutive active version of IkB. This indicates that overactivity of TNFα in macrophages inhibits fin regeneration. In agreement with results from knockdown of *tnfrsf1a* function by morpholino oligonucleotides^31^ we note that loss of TNFα activity results in a reduced proliferative response. Based on our results and others, we therefore hypothesise that it is critical to control the level of TNFα signalling to regulate the balance between proliferation and differentiation in the wound blastema for the resolution of inflammation and tissue repair.

A switch from a pro to an anti-inflammatory or resolution state is critical for effective wound repair and is impaired in chronic diseases such as muscle dystrophies. Such diseases often show a mixed inflammatory environment with elevated activity of both pro- and anti-inflammatory signals. Repair of the spinal cord and tailfin is impaired in zebrafish larvae lacking TNFα function, which occurs primarily in macrophages^55^.

We noticed that the anti-inflammatory cytokine *il10* is also up-regulated in response to IKK2 inhibition. Although IL10 is bound by p50 dimers and requires NF-kB activity in macrophages, it is also regulated by C/EBPbeta through TNFα activation of TAK1, leading to MAPK signalling^56,57^. Modelling of macrophage responses to LPS activation highlighted the potential role for TNFα to not only activate NF-kB but also IL10 through C/EBPbeta. One outcome of inhibiting IKK2 may be preferential activation of MKK3/6 by TAK1, leading to increased IL10 expression^58^.

In contrast to *tnfa*, the important pro-inflammatory gene *il1b* was down-regulated in response to NF-kB inhibition. This differs from regeneration experiments in which macrophages were ablated^31^ or are absent in *irf8* mutants, in which *il1b* shows a persistent and prolonged expression, concomitant with neutrophil retention. NF-kB inhibition does not alter neutrophil recruitment to the wound site, indicative of an il1b-independent recruitment process. Inhibition of NF-kB, therefore, does not prevent recruitment of inflammatory cells to tissue injury but does alter the cytokine profile in the wound blastema. This aberrant profile was observed at both 3 and 6 hpa, indicating that inhibition of NF-kB prevents a switch from a pro- to an anti-inflammatory environment, resulting in a mixed, non-polarised state. Tracking of macrophages expressing a mpeg:Dendra2 transgene when responding to tissue injury reveals differences in those showing a peripheral distribution compared to those in the caudal haematopoietic tissue (CHT) of larvae^59^. The peripheral macrophages perdured at the injury longer than those recruited from the circulation and were required for reducing reactive oxygen species and *il1b* expression. In our time-lapse movies, we could not distinguish whether macrophages arising from the CHT or periphery showed differential expression of *tnfa*, but as most macrophages recruited to the injury eventually showed *tnfa* expression can assume this included both peripheral and CHT-derived macrophages. The implication of this is that NF-kB acts as a rheostat to control TNFα activity in macrophages responding to injury and hence modulates the local inflammatory response.

Although NF-kB is described as principally driving inflammation, our results showing it regulates TNFα to modulate local inflammation is in agreement with a number of studies highlighting the importance of NF-kB signalling for promoting resolution of inflammation^60,61^. We observe different responses to inhibition of NF-kB by two important cytokines associated with inflammation—*il1b* in epidermis and the wound blastema and by *tnfa* in macrophages. Our analysis of cell behaviour implies that an auto-regulatory feedback loop mediated by TNFα regulates the initial pro-inflammatory response of macrophages to damage signals such as prostaglandins, HMGB1 and release of nuclear DNA. Subsequent recruitment of neutrophils and macrophages results in the release of pro-inflammatory signals that promote cell proliferation at the wound blastema. These signals include IL1 and TNFα and it is often assumed in models of wound healing that these signals are released by the inflammatory cells to regulate the wound blastema and recruit or polarise other immune cells. It is striking that a loss of *il1b* but not *tnfa* in macrophages, due to inhibition of NF-kB, results in an aberrant proliferation response in the wound blastema. Elevated *il1b* in *hai* and *cloche* mutants or by overexpression was demonstrated to induce excessive progenitor proliferation at the injury site^62^. We observe excess proliferation in the presence of *tnfa* overexpression but with a loss of *il1b*, indicating that elevated activity of TNFα at the injury alters proliferation. Similar manipulations by injection of TNFα or indirectly, by bacterial infection, reveal that excess *tnfa* not only induces excess proliferation but also affects differentiation^54,63^ and we show this requires NF-kB activity in macrophages to regulate this. The aberrant inflammatory profile of the wound tissue when NF-kB activity is reduced resembles that observed in a number of chronic inflammatory conditions, such as Duchenne’s muscle dystrophy or COPD. Potentially, the phenotypes observed as a loss of NF-kB activity in macrophages reflect a chronic non-healing state typical for such diseases.

Our results reveal that TNFα expression is unexpectedly repressed or attenuated by NF-kB in macrophages in the context of an injury. Given the complexity of the feedback mechanisms involved in maintaining inflammatory signalling, this may represent a failsafe mechanism to ensure NF-kB modulates TNFα action regardless of inputs from other pathways. Decoupling these pathways using inhibitors of NF-kB causes loss of this rheostat function, enabling ramping up of TNFα expression in response to other inflammatory-inducing cues. Interestingly, we note that other cytokines are not regulated in a similar manner—neither *il1b* or *il6* is strongly inhibited by loss of NF-kB signalling. It would be intriguing to dissect the relative inputs controlling TNFα in macrophages and determine which NF-kB-regulated genes are important for regulating TNFα expression. Determining how complex inputs are interpreted by inflammatory cells in vivo is critical for understanding how immunomodulatory molecules become dysregulated in disease. Our discovery that NF-kB activity can be decoupled from *tnfa* expression in activated macrophages is important for considering interventions to inhibit NF-kB in the context of disease. Our results suggest that immune responses to injury in the context of NF-kB inhibition will over-activate TNFα, but will not induce a classic inflammatory profile, with implications for wound healing and clearing infection.

## Methods

### Animals and experimental procedures

Zebrafish were bred and maintained at the King’s College London Zebrafish Facility. Experiments using animals were performed in accordance with the U.K. Animals (Scientific Procedure) Act 1986 and the European Union animal welfare Directive 2010/63/EU under project license PPL PBC5F9B13. Embryos were obtained by natural spawning and embryonic fish were maintained in E3 medium at 28.5°C. After 5 dpf, larvae were fed with Gemma75 (Skretting) daily. Previously published wild-type strain AB and transgenic lines *TgBAC(csfr1a:GAL4-VP16)i186Tg*; *Tg(UAS-E1B:NTR-mCherry)i149Tg* (described as fms:gal4; UAS:NfsB-mCherry in this work)^64^, *Tg(hsp70l:GAL4-VP16)vu22g*^65^, *TgBAC(tnfa:egfp)pd1028Tg*^26^, *Tg(6xHsa.NFKB:EGFP)nc1Tg*^66^ were used. A *tnfrsf1a* mutant (sa8496) with a point mutation in exon three was identified from an ENU mutagenesis screen^67^. We generated a *Tg(UAS:IkBA-SR;cmlc:mCherry)ed200Tg* line by co-injecting plasmid DNA pBH-UAS:IkB-SR-cmlc:mCherry with tol2 transposase RNA into single-cell staged embryos and selecting for animals expressing mCherry in the heart. F0 founders were outcrossed with wild-type zebrafish, F1 animals with mCherry-positive hearts were selected to grow up. F1 carriers were outcrossed with wild-type fish again. A F2 generation of carriers with a single insertion was taken forward to establish the *Tg(UAS:IKB-SR; cmlc:mCherry)ed200Tg* line.

Injuries were performed on 2 dpf larvae by transecting the tailfin posterior to the notochord using a sharp scalpel in the presence of anaesthetic (0.004% w/v tricaine methanesulphonate, MS-222). Larvae were exposed to compounds BMS-345541 (Sigma) at 2.5 mM, JSH-23 (Sigma) at 300 µM and pentoxifylline (Sigma) at 70 µM dissolved in embryo media E3. For imaging of cell behaviour, larvae were anaesthetised and immobilised in low-melting-point agarose (1.5%, Sigma). CRISPR mutagenesis of *tnfa* was performed by injecting gRNAs designed to *tnfa* together with Cas9 protein into 1-cell stage embryos as described previously^55^. CRISPR mutagenesis of *tnfrsf1b* was performed using gRNAs designed to exon 1 with sequences 5′-AGTGTGCACCGTGTCCAACT-3′, 5′-AAGTGTGCACCGTGTCCAAC-3′, 5′-CGGTCTGAAAACGTCCCAGT-3′; gRNAs were injected as for *tnfa* and mutagenesis was confirmed by sequencing of *n* = 20 larvae.

### Immunolabelling and in situ hybridisation

Larvae were euthanised with 0.02% w/v Tricaine and fixed in 4% w/v paraformaldehyde (PFA) overnight at 4 °C followed by permeabilization and incubation with primary antibodies to p65/ RelA (Cell Signalling Technology), eGFP (Millipore) or mCherry (Takara) as appropriate.

BrdU incorporation was performed by exposing larvae to 10 mM BrdU (Sigma) in E3, followed by fixation in 4% PFA, immersion in 2 M HCl for 1 h, then washed in buffer and detected with rat anti-BrdU (Abcam). Secondary antibodies used were Alexa-conjugated antibodies diluted 1:500 in goat serum and PBT.

In situ hybridisation was performed as described previously^68^ using digoxygenin-labelled anti-sense riboprobes to *tnfa*, *i1lb*, *nfikbaa* (ikbaa) and *nfikbab* (ikbab).

### RNA isolation and qRT-PCR

Total RNA was isolated from larval tissues using TriReagent (Sigma) and 500 ng reverse transcribed into cDNA using random hexamer primers (Promega) by M-MLV (Promega). Quantitative PCR was performed using a BioRad CFX384 Thermal Cycler with qPCRBIO SyGreen Lo-Rox mix (PCR BioSystems). Manager software (BioRad). Relative expression was calculated by comparing ratiometric changes of target genes relative to *eF1a* after correcting for primer efficiency using the REST package^69^. Significance of the difference between control and experimental relative expression is calculated using a pairwise fixed reallocation randomisation test.

### Molecular cloning

A tol2-based zebrafish transgenic plasmid was generated for overexpression of a constitutive active human Inhibitor of kappaB alpha (IKBA) under the control of a UAS promoter. Human IKBA carrying mutations S32A and S36A (gift from Neil Perkins^70^) was cloned into a bleeding heart construct pBH-UAS-mcs-YFP (gift from Dr. Michael Nonet), by replacing YFP between AgeI and ClaI sites to create a plasmid containing tol2-cmlc2:mCherry-UAS:IKBASRS32A/S36A-tol2 (pUAS:IkBA-SR).

### Image acquisition and analysis

Images of live samples were acquired on either a Zeiss LSM7MP multiphoton microscope using a 25× water dipping objective (NA = 1.0) or a Zeiss LSM880. Confocal imaging was performed using a Leica SP5 confocal using a 20x air objective (NA = 0.75) or 40× oil immersion objective (NA = 0.25). Confocal images were analysed in Fiji. Time-lapsed movies were analysed using Imaris 9.0 (Bitplane) using the Spot function for identifying and tracking macrophages.

### Statistical analysis

All statistical analysis was performed using Prism (Graphpad). Data was assessed for normality and variance using Shapiro-Wilk and Kolmogorov-Smirnov tests. Units were defined as individual cells in different animals. Depending on normality and variance, the significance of difference between conditions was tested by unpaired Student’s *t*-test, 1-way ANOVA with Dunnett’s test for multiple comparisons, 2-way ANOVA with Tukey’s test for multiple comparisons, or by a Sidak’s multiple comparison test to identify the significance of difference across conditions. Comparisons of cell behaviour between conditions were performed by treating each measurement of cell speed or directionality for a time series as a single datapoint. Analysis of time-lapsed data was performed by blinding file names to avoid bias. Comparisons of tail length were tested for differences over time.

## Supplementary information

Supplementary information

Supplementary movie1

Supplementary movie2

Supplementary movie3

Supplementary movie4

Supplementary movie5

Supplementary movie6

Supplementary movie7

Supplementary movie8

Supplementary movie9

Supplementary movie10

Supplementary movie11

Supplementary movie12

Supplementary movie13

Supplementary movie14

Supplementary movie15

Supplementary movie16

Supplementary movie17

Supplementary movie18

Supplementary movie19

Supplementary movie20

## Data Availability

All quantification of cell numbers and cell trajectories from time-lapsed movies are provided in the supplementary information as tables of data. Ratiometric values for REST analyses of qPCR experiments are provided as supplementary tables of data. Projections of representative time-lapse movies for all experiments are included as supplementary movies. Access to raw movie data can be requested by contacting the corresponding author.

## References

[1] Albrecht, H., Schook, L. B. & Jongeneel, C. V. Nuclear migration of NF-kappa B correlates with TNF-alpha mRNA accumulation. *J. Inflamm.* **45**, 64–71 (1995). 7583354

[2] Webster, J. D. & Vucic, D. The balance of TNF mediated pathways regulates inflammatory cell death signaling in healthy and diseased tissues. *Front. Cell Dev. Biol.* **8**, 365 (2020). 32671059 10.3389/fcell.2020.00365 PMC7326080

[3] Atretkhany, K. N., Gogoleva, V. S., Drutskaya, M. S. & Nedospasov, S. A. Distinct modes of TNF signaling through its two receptors in health and disease. *J. Leukoc. Biol.* **107**, 893–905 (2020). 32083339 10.1002/JLB.2MR0120-510R

[4] Biswas, S. K. & Lewis, C. E. NF-kappaB as a central regulator of macrophage function in tumors. *J. Leukoc. Biol.* **88**, 877–884 (2010). 20573802 10.1189/jlb.0310153

[5] Mills, C. D., Kincaid, K., Alt, J. M., Heilman, M. J. & Hill, A. M. M-1/M-2 macrophages and the Th1/Th2 paradigm. *J. Immunol.* **164**, 6166–6173 (2000). 28923981 10.4049/jimmunol.1701141

[6] Wang, N., Liang, H. & Zen, K. Molecular mechanisms that influence the macrophage m1-m2 polarization balance. *Front. Immunol.* **5**, 614 (2014). 25506346 10.3389/fimmu.2014.00614 PMC4246889

[7] Williams, R. A., Timmis, J. & Qwarnstrom, E. E. Computational models of the NF-KB signalling pathway. *Computation* **2**, 131–158 (2014).

[8] Burke, J. R. et al. BMS-345541 is a highly selective inhibitor of I kappa B kinase that binds at an allosteric site of the enzyme and blocks NF-kappa B-dependent transcription in mice. *J. Biol. Chem.* **278**, 1450–1456 (2003). 12403772 10.1074/jbc.M209677200

[9] Jobin, C. et al. Inhibition of proinflammatory molecule production by adenovirus-mediated expression of a nuclear factor kappaB super-repressor in human intestinal epithelial cells. *J. Immunol.* **160**, 410–418 (1998). 9551998

[10] Bondeson, J., Foxwell, B., Brennan, F. & Feldmann, M. Defining therapeutic targets by using adenovirus: blocking NF-kappaB inhibits both inflammatory and destructive mechanisms in rheumatoid synovium but spares anti-inflammatory mediators. *Proc. Natl. Acad. Sci. USA* **96**, 5668–5673 (1999). 10318942 10.1073/pnas.96.10.5668 PMC21918

[11] Enyedi, B., Kala, S., Nikolich-Zugich, T. & Niethammer, P. Tissue damage detection by osmotic surveillance. *Nat. Cell Biol.* **15**, 1123–1130 (2013). 23934216 10.1038/ncb2818 PMC3826879

[12] Niethammer, P., Grabher, C., Look, A. T. & Mitchison, T. J. A tissue-scale gradient of hydrogen peroxide mediates rapid wound detection in zebrafish. *Nature* **459**, 996–999 (2009). 19494811 10.1038/nature08119 PMC2803098

[13] Stuehr, D. J. & Marletta, M. A. Mammalian nitrate biosynthesis: mouse macrophages produce nitrite and nitrate in response to Escherichia coli lipopolysaccharide. *Proc. Natl. Acad. Sci. USA* **82**, 7738–7742 (1985). 3906650 10.1073/pnas.82.22.7738 PMC391409

[14] Tauzin, S., Starnes, T. W., Becker, F. B., Lam, P. Y. & Huttenlocher, A. Redox and Src family kinase signaling control leukocyte wound attraction and neutrophil reverse migration. *J. Cell Biol.* **207**, 589–598 (2014). 25488917 10.1083/jcb.201408090 PMC4259815

[15] Venereau, E. et al. Mutually exclusive redox forms of HMGB1 promote cell recruitment or proinflammatory cytokine release. *J. Exp. Med.* **209**, 1519–1528 (2012). 22869893 10.1084/jem.20120189 PMC3428943

[16] Chase, A. J., Bond, M., Crook, M. F. & Newby, A. C. Role of nuclear factor-kappa B activation in metalloproteinase-1, -3, and -9 secretion by human macrophages in vitro and rabbit foam cells produced in vivo. *Arterioscler. Thromb. Vasc. Biol.* **22**, 765–771 (2002). 12006388 10.1161/01.atv.0000015078.09208.92

[17] Rhee, J. W. et al. NF-kappaB-dependent regulation of matrix metalloproteinase-9 gene expression by lipopolysaccharide in a macrophage cell line RAW 264.7. *J. Biochem. Mol. Biol.* **40**, 88–94 (2007). 17244487 10.5483/bmbrep.2007.40.1.088

[18] Kew, R. R., Penzo, M., Habiel, D. M. & Marcu, K. B. The IKKalpha-dependent NF-kappaB p52/RelB noncanonical pathway is essential to sustain a CXCL12 autocrine loop in cells migrating in response to HMGB1. *J. Immunol.* **188**, 2380–2386 (2012). 22287708 10.4049/jimmunol.1102454 PMC3288724

[19] Penzo, M., Habiel, D. M., Ramadass, M., Kew, R. R. & Marcu, K. B. Cell migration to CXCL12 requires simultaneous IKKalpha and IKKbeta-dependent NF-kappaB signaling. *Biochim. Biophys. Acta* **1843**, 1796–1804 (2014). 24747690 10.1016/j.bbamcr.2014.04.011 PMC4096130

[20] Ratheesh, A. et al. Drosophila TNF modulates tissue tension in the embryo to facilitate macrophage invasive migration. *Dev. Cell* **45**, 331–346.e337 (2018). 29738712 10.1016/j.devcel.2018.04.002

[21] Choe, J. Y., Hun Kim, J., Park, K. Y., Choi, C. H. & Kim, S. K. Activation of dickkopf-1 and focal adhesion kinase pathway by tumour necrosis factor alpha induces enhanced migration of fibroblast-like synoviocytes in rheumatoid arthritis. *Rheumatology* **55**, 928–938 (2016). 26715774 10.1093/rheumatology/kev422

[22] Pokharel, S. M., Chiok, K., Shil, N. K., Mohanty, I. & Bose, S. Tumor Necrosis Factor-alpha utilizes MAPK/NFkappaB pathways to induce cholesterol-25 hydroxylase for amplifying pro-inflammatory response via 25-hydroxycholesterol-integrin-FAK pathway. *PLoS ONE* **16**, e0257576 (2021). 34551004 10.1371/journal.pone.0257576 PMC8457477

[23] Torrente, Y. et al. Tumor necrosis factor-alpha (TNF-alpha) stimulates chemotactic response in mouse myogenic cells. *Cell Transplant.* **12**, 91–100 (2003). 12693669 10.3727/000000003783985115

[24] Gurevich, D. B. et al. Live imaging of wound angiogenesis reveals macrophage orchestrated vessel sprouting and regression. *EMBO J* **37**, 10.15252/embj.201797786 (2018). 10.15252/embj.201797786 PMC6028026 29866703

[25] Nguyen-Chi, M. et al. Identification of polarized macrophage subsets in zebrafish. *eLife* **4**, e07288 (2015). 26154973 10.7554/eLife.07288 PMC4521581

[26] Marjoram, L. et al. Epigenetic control of intestinal barrier function and inflammation in zebrafish. *Proc. Natl. Acad. Sci. USA* **112**, 2770–2775 (2015). 25730872 10.1073/pnas.1424089112 PMC4352795

[27] Ratnayake, D. et al. Macrophages provide a transient muscle stem cell niche via NAMPT secretion. *Nature* **591**, 281–287 (2021). 33568815 10.1038/s41586-021-03199-7

[28] Shin, H. M. et al. Inhibitory action of novel aromatic diamine compound on lipopolysaccharide-induced nuclear translocation of NF-kappaB without affecting IkappaB degradation. *FEBS Lett.* **571**, 50–54 (2004). 15280016 10.1016/j.febslet.2004.06.056

[29] Daroczi, B., Kari, G., Ren, Q., Dicker, A. P. & Rodeck, U. Nuclear factor kappaB inhibitors alleviate and the proteasome inhibitor PS-341 exacerbates radiation toxicity in zebrafish embryos. *Mol. Cancer Ther.* **8**, 2625–2634 (2009). 19723885 10.1158/1535-7163.MCT-09-0198 PMC2846641

[30] Kuri, P., Ellwanger, K., Kufer, T. A., Leptin, M. & Bajoghli, B. A high-sensitivity bi-directional reporter to monitor NF-kappaB activity in cell culture and zebrafish in real time. *J. Cell Sci.* **130**, 648–657 (2017). 27980067 10.1242/jcs.196485

[31] Nguyen-Chi, M. et al. TNF signaling and macrophages govern fin regeneration in zebrafish larvae. *Cell Death Dis.* **8**, e2979 (2017). 28796253 10.1038/cddis.2017.374 PMC5596562

[32] Karra, R., Knecht, A. K., Kikuchi, K. & Poss, K. D. Myocardial NF-kappaB activation is essential for zebrafish heart regeneration. *Proc. Natl. Acad. Sci. USA* **112**, 13255–13260 (2015). 26472034 10.1073/pnas.1511209112 PMC4629358

[33] Mishra, R., Sehring, I., Cederlund, M., Mulaw, M. & Weidinger, G. NF-kappaB signaling negatively regulates osteoblast dedifferentiation during zebrafish bone regeneration. *Dev. Cell* **52**, 167–182.e167 (2020). 31866203 10.1016/j.devcel.2019.11.016

[34] Mitchell, J. P. & Carmody, R. J. NF-kappaB and the transcriptional control of inflammation. *Int. Rev. Cell Mol. Biol.* **335**, 41–84 (2018). 29305014 10.1016/bs.ircmb.2017.07.007

[35] Shakhov, A. N., Collart, M. A., Vassalli, P., Nedospasov, S. A. & Jongeneel, C. V. Kappa B-type enhancers are involved in lipopolysaccharide-mediated transcriptional activation of the tumor necrosis factor alpha gene in primary macrophages. *J. Exp. Med.* **171**, 35–47 (1990). 2104921 10.1084/jem.171.1.35 PMC2187654

[36] Tian, B., Nowak, D. E., Jamaluddin, M., Wang, S. & Brasier, A. R. Identification of direct genomic targets downstream of the nuclear factor-kappaB transcription factor mediating tumor necrosis factor signaling. *J. Biol. Chem.* **280**, 17435–17448 (2005). 15722553 10.1074/jbc.M500437200

[37] Zhang, Q., Lenardo, M. J. & Baltimore, D. 30 years of NF-kappaB: a blossoming of relevance to human pathobiology. *Cell* **168**, 37–57 (2017). 28086098 10.1016/j.cell.2016.12.012 PMC5268070

[38] Kanters, E. et al. Inhibition of NF-kappaB activation in macrophages increases atherosclerosis in LDL receptor-deficient mice. *J. Clin. Investig.* **112**, 1176–1185 (2003). 14561702 10.1172/JCI18580 PMC213488

[39] Lane, K. et al. Measuring signaling and RNA-seq in the same cell links gene expression to dynamic patterns of NF-kappaB activation. *Cell Syst.* **4**, 458–469.e455 (2017). 28396000 10.1016/j.cels.2017.03.010 PMC6748049

[40] Pekalski, J. et al. Spontaneous NF-kappaB activation by autocrine TNFα signaling: a computational analysis. *PLoS ONE* **8**, e78887 (2013). 24324544 10.1371/journal.pone.0078887 PMC3855823

[41] Sakai, J. et al. Lipopolysaccharide-induced NF-kappaB nuclear translocation is primarily dependent on MyD88, but TNFα expression requires TRIF and MyD88. *Sci. Rep.* **7**, 1428 (2017). 28469251 10.1038/s41598-017-01600-y PMC5431130

[42] Lee, T. K. et al. A noisy paracrine signal determines the cellular NF-kappaB response to lipopolysaccharide. *Sci. Signal.* **2**, ra65 (2009). 19843957 10.1126/scisignal.2000599 PMC2778577

[43] Gottschalk, R. A. et al. Distinct NF-kappaB and MAPK activation thresholds uncouple steady-state microbe sensing from anti-pathogen inflammatory responses. *Cell Syst.* **2**, 378–390 (2016). 27237739 10.1016/j.cels.2016.04.016 PMC4919147

[44] Guma, M. et al. Constitutive intestinal NF-kappaB does not trigger destructive inflammation unless accompanied by MAPK activation. *J. Exp. Med.* **208**, 1889–1900 (2011). 21825016 10.1084/jem.20110242 PMC3171091

[45] Wang, A. G., Son, M., Kenna, E., Thom, N. & Tay, S. NF-kappaB memory coordinates transcriptional responses to dynamic inflammatory stimuli. *Cell Rep.* **40**, 111159 (2022). 35977475 10.1016/j.celrep.2022.111159 PMC10794069

[46] Parameswaran, N. & Patial, S. Tumor necrosis factor-alpha signaling in macrophages. *Crit. Rev. Eukaryot. Gene Expr.* **20**, 87–103 (2010). 21133840 10.1615/critreveukargeneexpr.v20.i2.10 PMC3066460

[47] Perez, C. et al. A nonsecretable cell surface mutant of tumor necrosis factor (TNF) kills by cell-to-cell contact. *Cell* **63**, 251–258 (1990). 2208285 10.1016/0092-8674(90)90158-b

[48] Richter, C. et al. The tumor necrosis factor receptor stalk regions define responsiveness to soluble versus membrane-bound ligand. *Mol. Cell. Biol.* **32**, 2515–2529 (2012). 22547679 10.1128/MCB.06458-11 PMC3434479

[49] Schlaepfer, D. D. et al. Tumor necrosis factor-alpha stimulates focal adhesion kinase activity required for mitogen-activated kinase-associated interleukin 6 expression. *J. Biol. Chem.* **282**, 17450–17459 (2007). 17438336 10.1074/jbc.M610672200

[50] Mitra, A. et al. Cell geometry dictates TNFα-induced genome response. *Proc. Natl. Acad. Sci. USA* **114**, E3882–E3891 (2017). 28461498 10.1073/pnas.1618007114 PMC5441774

[51] Barros-Becker, F., Lam, P. Y., Fisher, R. & Huttenlocher, A. Live imaging reveals distinct modes of neutrophil and macrophage migration within interstitial tissues. *J. Cell Sci.* **130**, 3801–3808 (2017). 28972134 10.1242/jcs.206128 PMC5702045

[52] Linehan, J. B., Lucas Zepeda, J., Mitchell, T. A. & LeClair, E. E. Follow that cell: Leukocyte migration in L-plastin mutant zebrafish. *Cytoskeleton* **79**, 26–37 (2022). 35811499 10.1002/cm.21717

[53] Travnickova, J. et al. Macrophage morphological plasticity and migration is Rac signalling and MMP9 dependant. *Sci. Rep.* **11**, 10123 (2021). 33980872 10.1038/s41598-021-88961-7 PMC8115330

[54] Miskolci, V. et al. Distinct inflammatory and wound healing responses to complex caudal fin injuries of larval zebrafish. *eLife* **8**, 10.7554/eLife.45976 (2019). 10.7554/eLife.45976 PMC6602581 31259685

[55] Tsarouchas, T. M. et al. Dynamic control of proinflammatory cytokines Il-1beta and Tnf-alpha by macrophages in zebrafish spinal cord regeneration. *Nat. Commun.* **9**, 4670 (2018). 30405119 10.1038/s41467-018-07036-w PMC6220182

[56] Chakrabarti, A. et al. Protein kinase R-dependent regulation of interleukin-10 in response to double-stranded RNA. *J. Biol. Chem.* **283**, 25132–25139 (2008). 18625702 10.1074/jbc.M804770200 PMC2533086

[57] Csoka, B. et al. A2A adenosine receptors and C/EBPbeta are crucially required for IL-10 production by macrophages exposed to Escherichia coli. *Blood* **110**, 2685–2695 (2007). 17525287 10.1182/blood-2007-01-065870 PMC1988939

[58] Tomaiuolo, M., Kottke, M., Matheny, R. W., Reifman, J. & Mitrophanov, A. Y. Computational identification and analysis of signaling subnetworks with distinct functional roles in the regulation of TNF production. *Mol. Biosyst.* **12**, 826–838 (2016). 26751842 10.1039/c5mb00456j

[59] Morales, R. A. & Allende, M. L. Peripheral macrophages promote tissue regeneration in zebrafish by fine-tuning the inflammatory response. *Front. Immunol.* **10**, 253 (2019). 30891030 10.3389/fimmu.2019.00253 PMC6413720

[60] de Jesus, T. J. & Ramakrishnan, P. NF-kappaB c-Rel dictates the inflammatory threshold by acting as a transcriptional repressor. *iScience* **23**, 100876 (2020). 32062419 10.1016/j.isci.2020.100876 PMC7031323

[61] Lawrence, T., Bebien, M., Liu, G. Y., Nizet, V. & Karin, M. IKKalpha limits macrophage NF-kappaB activation and contributes to the resolution of inflammation. *Nature* **434**, 1138–1143 (2005). 15858576 10.1038/nature03491

[62] Hasegawa, T. et al. Transient inflammatory response mediated by interleukin-1beta is required for proper regeneration in zebrafish fin fold. *eLife* **6**, e22716 (2017). 28229859 10.7554/eLife.22716 PMC5360449

[63] Nguyen-Chi, M. et al. Pro-resolving mediator protectin D1 promotes epimorphic regeneration by controlling immune cell function in vertebrates. *Br. J. Pharmacol.* **177**, 4055–4073 (2020). 32520398 10.1111/bph.15156 PMC7429485

[64] Gray, C. et al. Simultaneous intravital imaging of macrophage and neutrophil behaviour during inflammation using a novel transgenic zebrafish. *Thromb. Haemost.* **105**, 811–819 (2011). 21225092 10.1160/TH10-08-0525

[65] Shin, J., Poling, J., Park, H. C. & Appel, B. Notch signaling regulates neural precursor allocation and binary neuronal fate decisions in zebrafish. *Development* **134**, 1911–1920 (2007). 17442701 10.1242/dev.001602

[66] Kanther, M. et al. Microbial colonization induces dynamic temporal and spatial patterns of NF-kappaB activation in the zebrafish digestive tract. *Gastroenterology* **141**, 197–207 (2011). 21439961 10.1053/j.gastro.2011.03.042 PMC3164861

[67] Kettleborough, R. N. et al. A systematic genome-wide analysis of zebrafish protein-coding gene function. *Nature* **496**, 494–497 (2013). 23594742 10.1038/nature11992 PMC3743023

[68] Thisse, B. & Thisse, C. In situ hybridization on whole-mount zebrafish embryos and young larvae. *Methods Mol. Biol.* **1211**, 53–67 (2014). 25218376 10.1007/978-1-4939-1459-3_5

[69] Pfaffl, M. W., Horgan, G. W. & Dempfle, L. Relative expression software tool (REST) for group-wise comparison and statistical analysis of relative expression results in real-time PCR. *Nucleic Acids Res.* **30**, e36 (2002). 11972351 10.1093/nar/30.9.e36 PMC113859

[70] Batra, R. K. et al. IkappaBalpha gene transfer is cytotoxic to squamous-cell lung cancer cells and sensitizes them to tumor necrosis factor-alpha-mediated cell death. *Am. J. Respir. Cell Mol. Biol.* **21**, 238–245 (1999). 10423407 10.1165/ajrcmb.21.2.3470

