# Supplementary material for: An NF-kB/TNF-alpha signalling feedback loop acts to coordinate tissue regeneration and macrophage behaviour in zebrafish
Source: NPJ Regen Med. 2025 Jun 3;10:27. doi: 10.1038/s41536-025-00414-1 (PMC12134371; doi:10.1038/s41536-025-00414-1)
Supplement: Supplementary file 1 — Supplementary information [file 41536_2025_414_MOESM1_ESM.pdf]

Supplementary Information

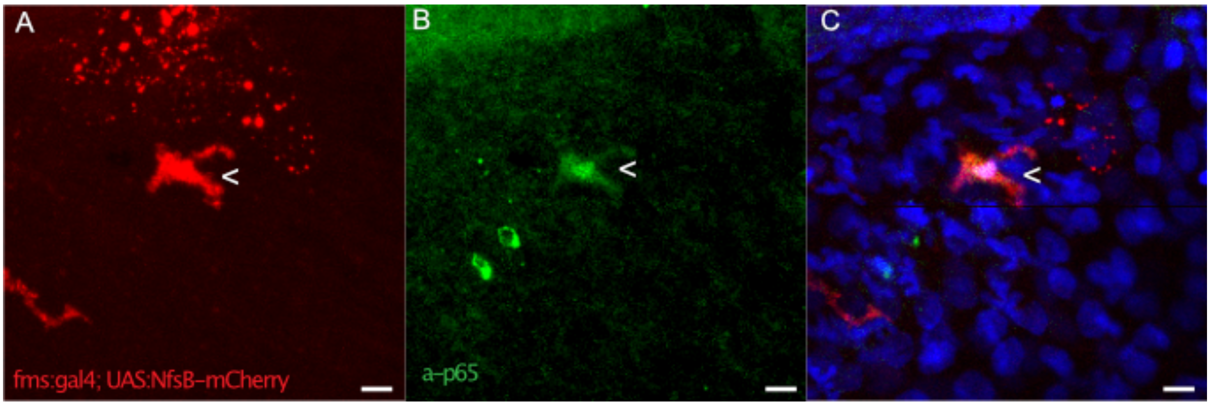

Supplementary Figure 1: macrophages show nuclear localised RelA/p65 in response to injury. Following tailfin amputation larvae were fixed and processed by immunolabelling to detect p65. Macrophages expressing fms:gal4; UAS:NfsB-mCherry (A) at the injury site show p65 labelling (B) which is nuclear localised (C, arrowhead).
Scale bars 10 µm (A-C).

A

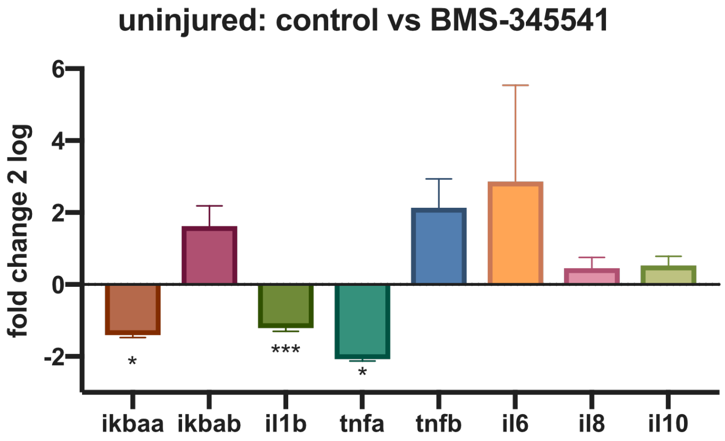

B

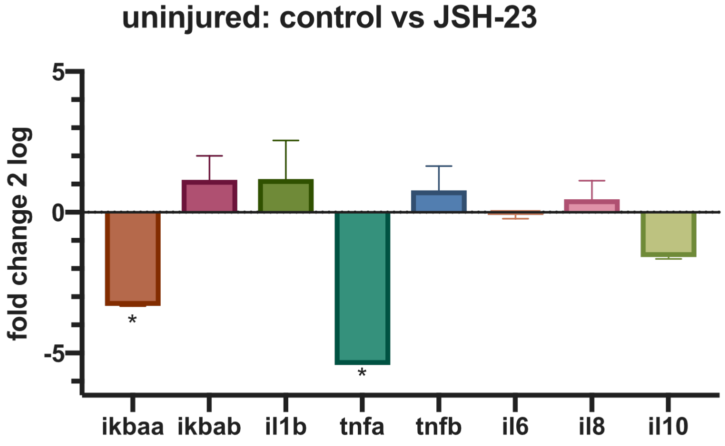

Supplementary Figure 2: treatment of uninjured larvae with NF-kB inhibitors results in reduced expression of NF-kB regulated genes. Uninjured 3 dpf larvae were treated with DMSO (control), 2.5 mM BMS-345541 or 300 µM JSH-23 for 3 hours (hpa, A) or 6 hours (B). Extracted RNA from pooled animals (n= 60 animals/ condition) was used as template for RT-qPCR and fold difference plotted for injured relative to uninjured animals for each condition.
Significance for fold change was calculated using the REST software after correcting for primer efficiency. Significance for differences between groups are shown ($p<0.001$ ***, $p<0.01$ **, $p<0.05$ *, not significant n.s.). Bar plots show mean values with bars representing standard error of the mean.

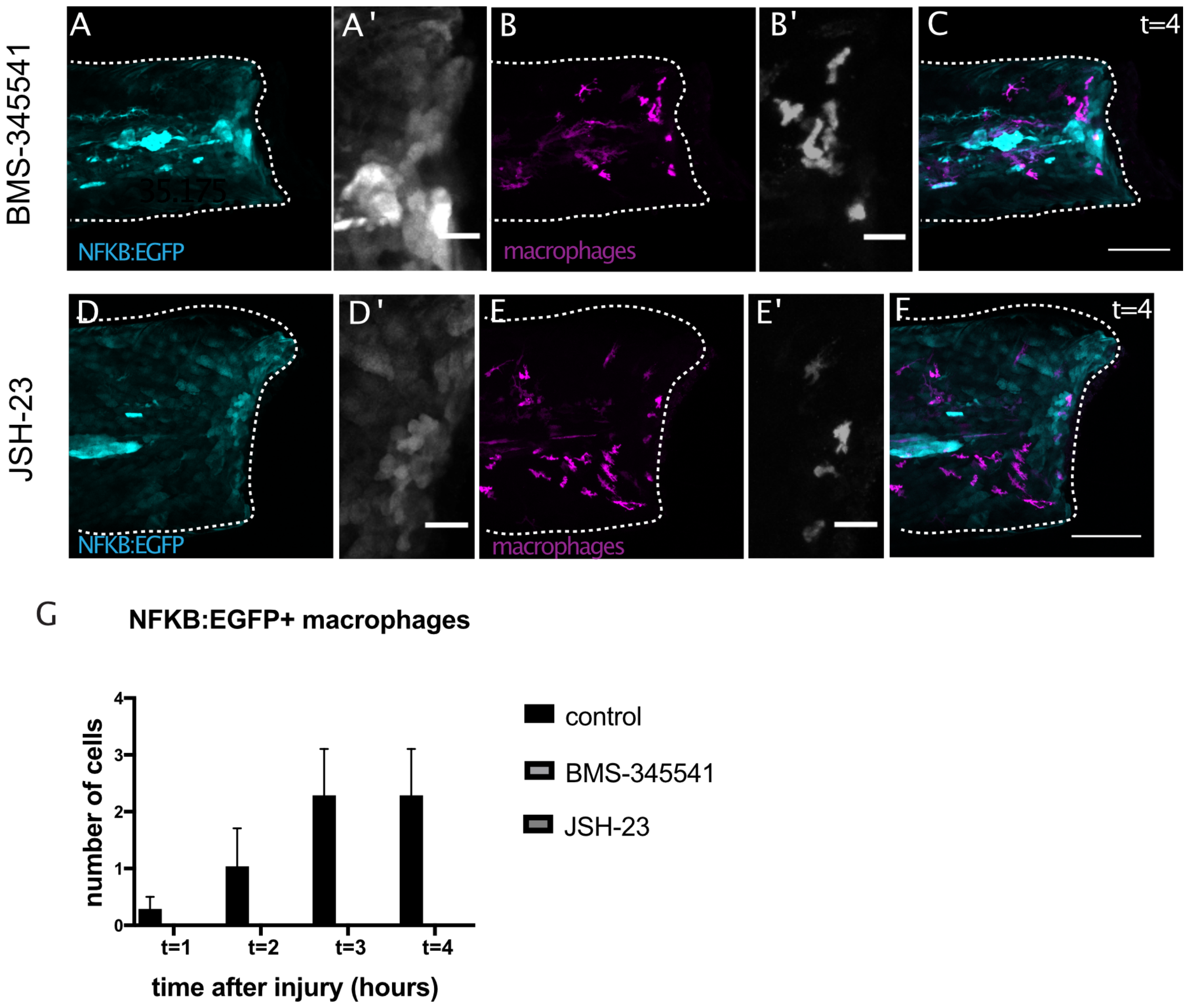

Supplementary Figure 3: NFKB:EGFP reporter activity is lost in macrophages in the presence of NF-kB inhibitors BMS-345541 or JSH-23. Tailfin injuries were performed on transgenic larvae expressing NFKB:EGFP reporter and animals exposed to 2.5 mM BMS-345541 (A-C) or 300 μM JSH-23 (D-F). Animals were observed by time-lapsed microscopy and macrophages expressing NFKB:EGFP visualised at 1 or 4 hpa then quantified (G, n = 4 animals per condition). Bar plots show mean values with bars representing standard error of the mean.

Scale bars 100 μm (A-D).

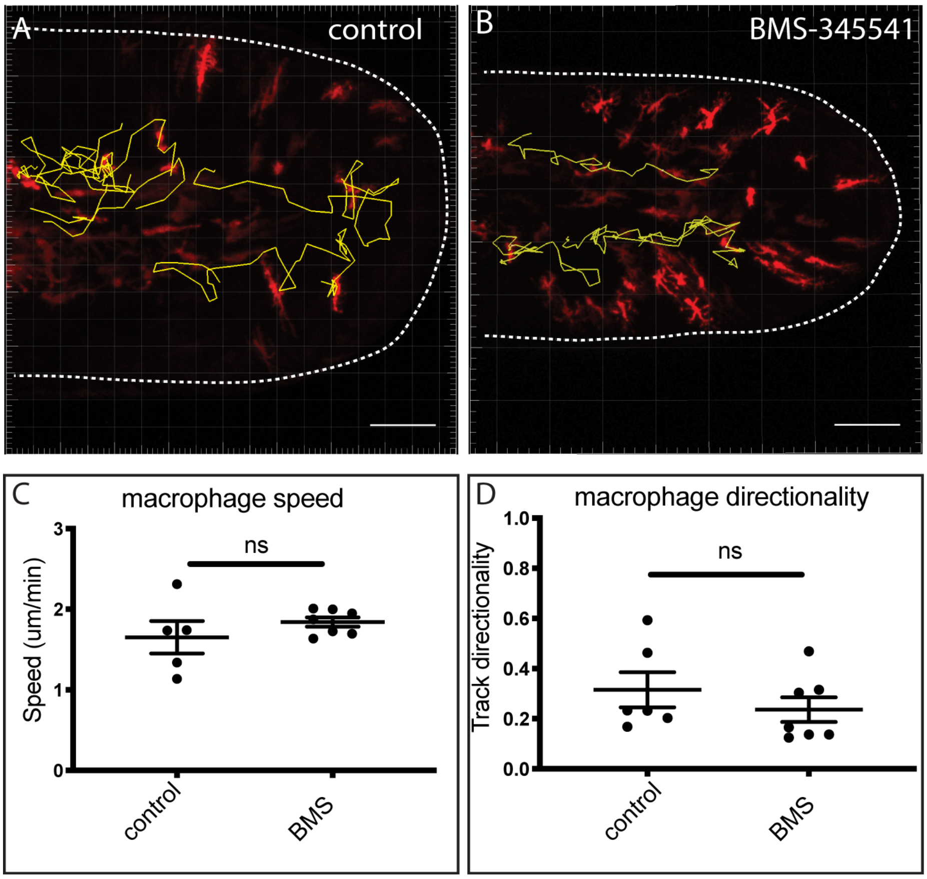

Supplementary Figure 4: macrophage behaviour is unaltered in response to inhibition of NF-kB activity in an absence of injury. Uninjured 3 dpf larvae expressing fms:gal4; UAS:NfsB-mCherry were treated with DMSO (control) or 2.5 mM BMS-345541. Macrophages patrolling the fin fold (outlined) were observed by time-lapsed microscopy for 4 hours and tracked using Imaris (yellow lines) to generate measures of instantaneous speed and directionality (A-D). Significance of differences for speed and directionality between control and drug treated animals was tested by unpaired t-test (n = 3 animals /condition). Significance is shown ($p<0.005$ ***, $p<0.01$ **, $p<0.05$ *, not significant n.s.). Bar plots show mean values with bars representing standard error of the mean.
Scale bar 100 μm.

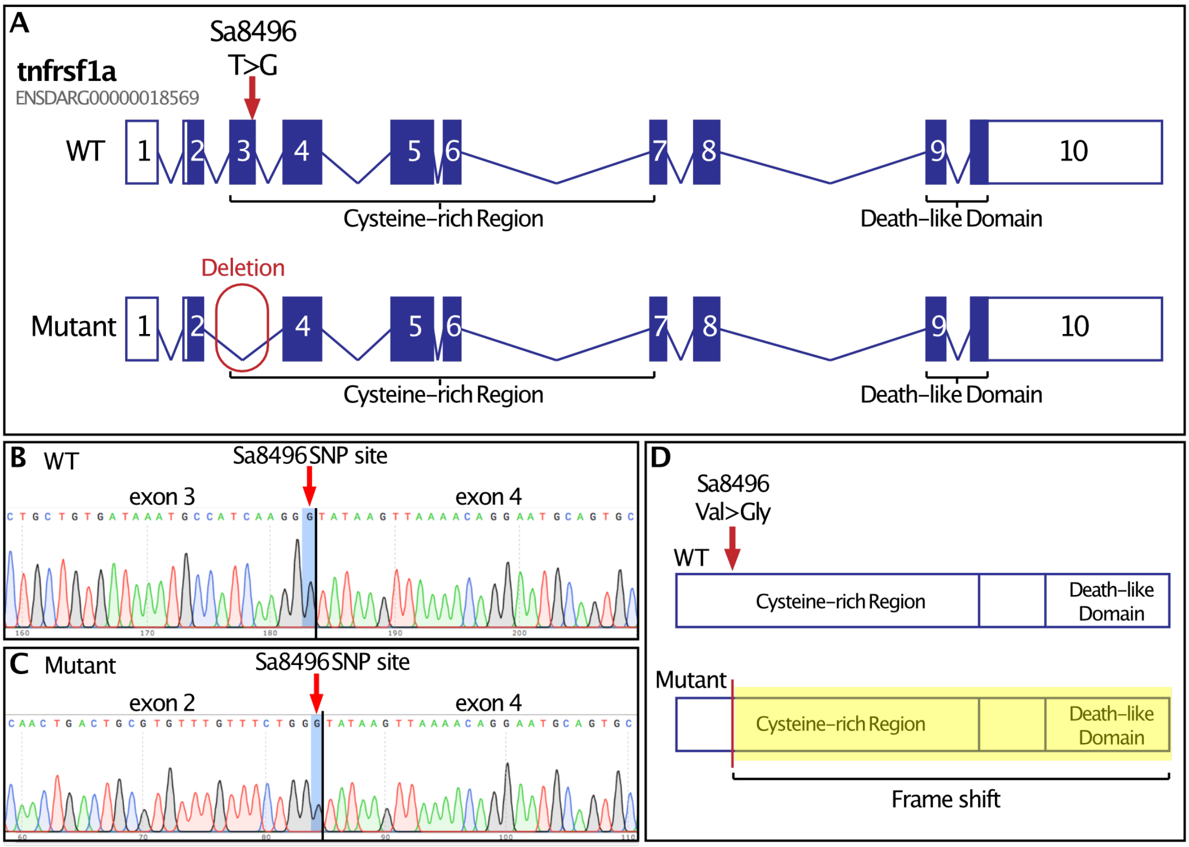

Supplementary Figure 5: schematic of the *tnfrsf1a* gene showing changes to splicing and consequences for protein translation for allele sa8496. *Tnfrsf1a* possesses 10 exons and encodes a 389 amino acid protein with a Cysteine-rich domain and a Death-like domain. A mutation of a splice donor site in exon 3 (T to G) results loss of the splice site in allele sa8496 (A). Sequencing of cDNA amplified from *tnfrsf1a* mutant and wildtype (WT) animals reveals altered splicing at this site results in loss of exon3 due to splicing from exon 2 (B, C). Changes to the open reading frame in sa8496 transcripts are predicted to result in a prematurely truncated protein lacking the Cysteine-rich region and Death-like domain (D).

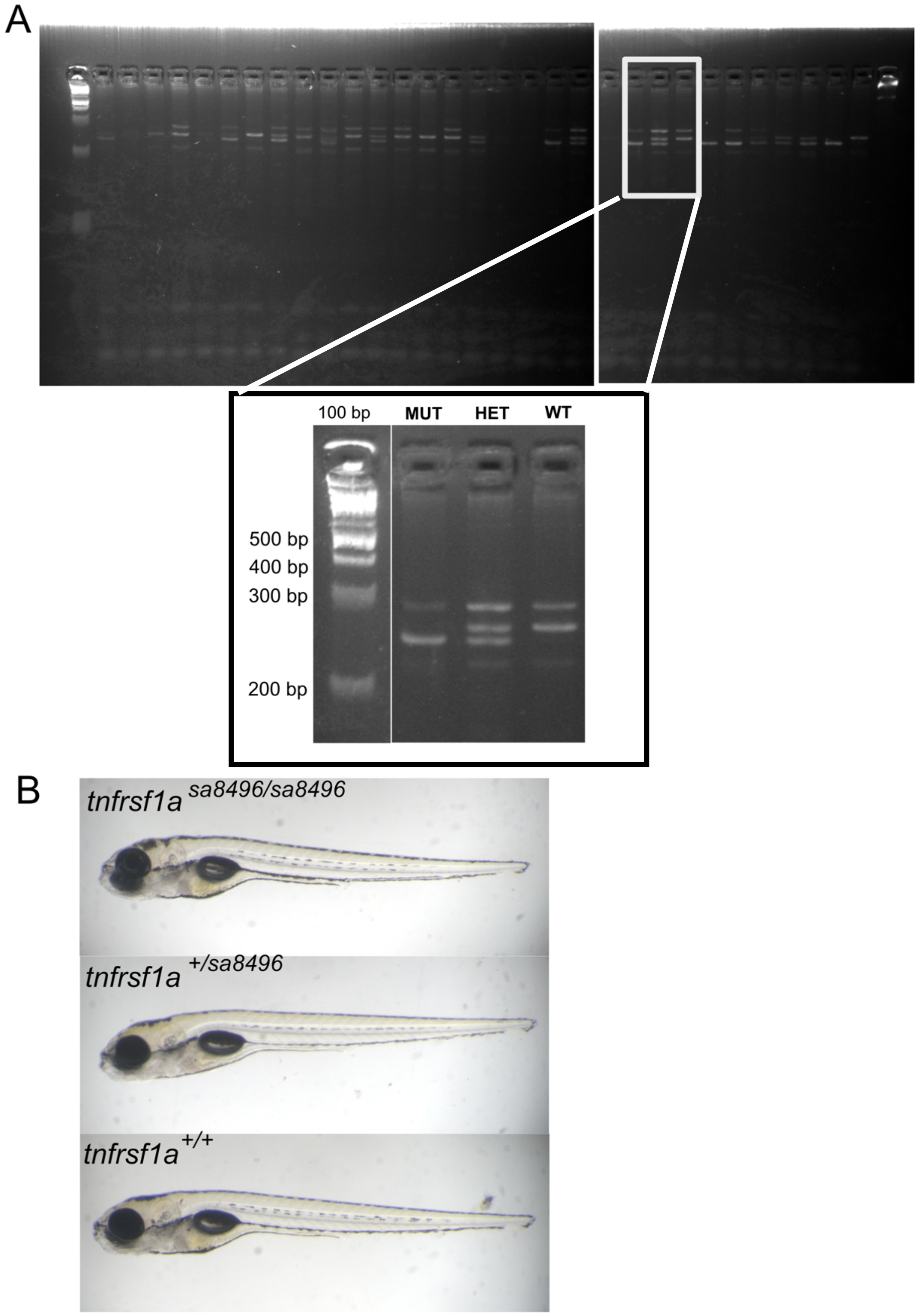

Supplementary Figure 6: characterisation of *tnfrsf1a (sa8496)* mutants. Amplification of *tnfrsf1a* transcripts from animals carrying the sa8496 allele by RT-PCR. Comparison of PCR products (boxed region) reveals altered splicing over the exon2/4 region results in a smaller product in mutant animals (MUT, 250 bp) compared to wildtype animals (WT, 290 bp), with heterozygote animals (HET) showing mixed transcript sizes (A). At 6 dpf *tnfrsf1a* mutant, heterozygote and wildtype siblings are phenotypically indistinguishable (B).

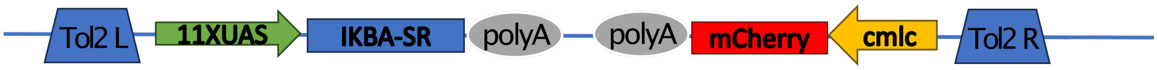

Supplementary Figure 7: A human super-repressor version of IkBA (S32A, S36A) was cloned into the bleeding heart construct pBH-UAS-mcs-YFP in place of YFP to create plasmid pT2-cmlc2:mCherry-UAS:IKBASR32A/S36A.

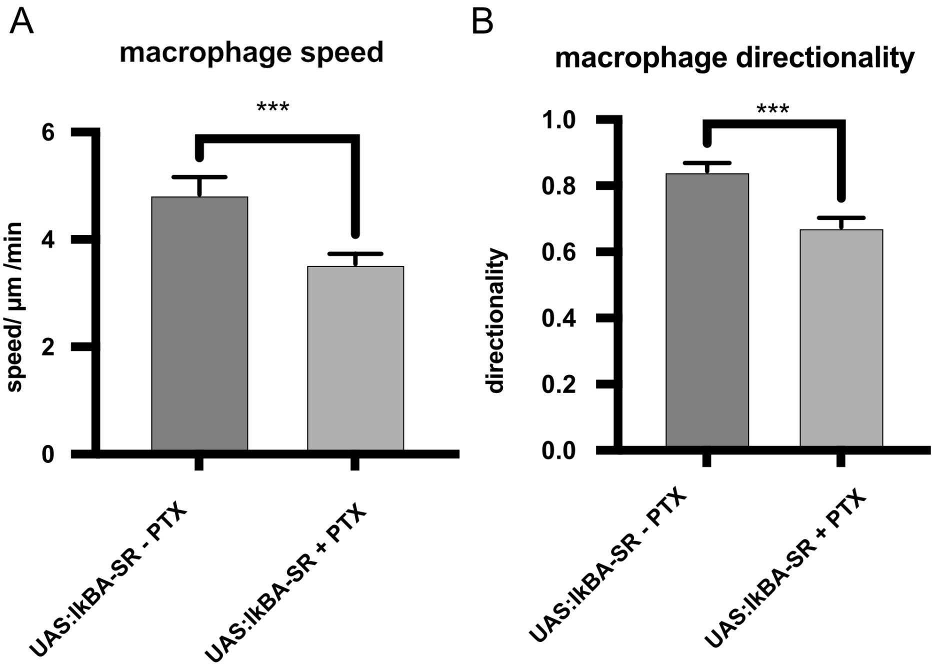

Supplementary Figure 8: Macrophage responses to injury in larvae over-expressing IkBA-SR in macrophages were attenuated by Pentoxyfilline (PTX) treatment. Cell instantaneous speed (A) and directionality (B) were measured from time-lapsed movies of transgenic fms:gal4; UAS:IkBA-SR larvae in the presence or absence of PTX following tail amputation. Significance of difference was tested by unpaired Student's t-test (n = 3 animals/ condition). Significance is shown (p<0.005 ***, p<0.01 **, p<0.05 *, not significant n.s.). Bar plots show mean values with bars representing standard error of the mean.

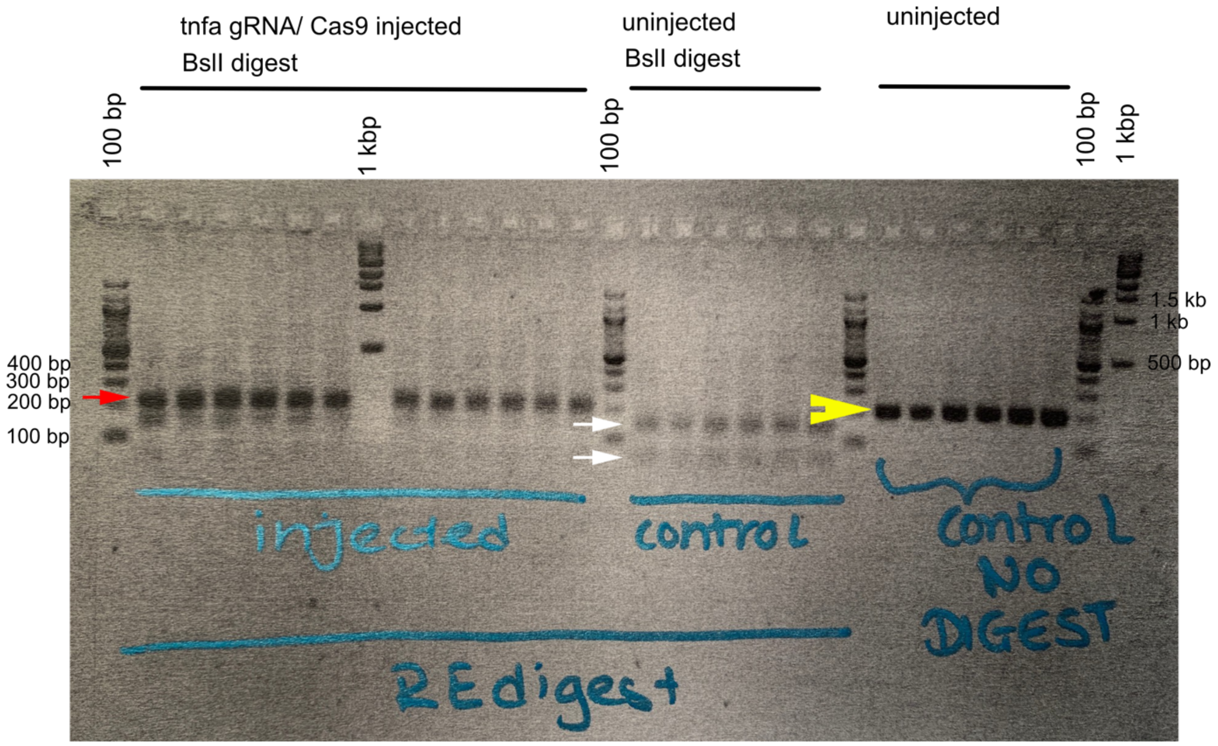

Supplementary Figure 9: Analysis of Cas9-mediated mutagenesis of *tnfa* in larvae. Genomic DNA was extracted from individual larvae that had been injected by gRNAs designed to tnfa and Cas9 protein or from uninjected control larvae following time-lapsed microscopy. A region of the *tnfa* locus was amplified by PCR to produce a 200 bp band (yellow arrows) and DNA products digested with *BslI* to discriminate between control (includes a *BslI* site, yellow arrows) and mutated (loss of *BslI* site, red arrow) sequences as per Tsarouchis et al (2018). Size markers are 100 bp DNA ladder (left) and 1 Kb ladder (right) with sizes shown for respective bands.

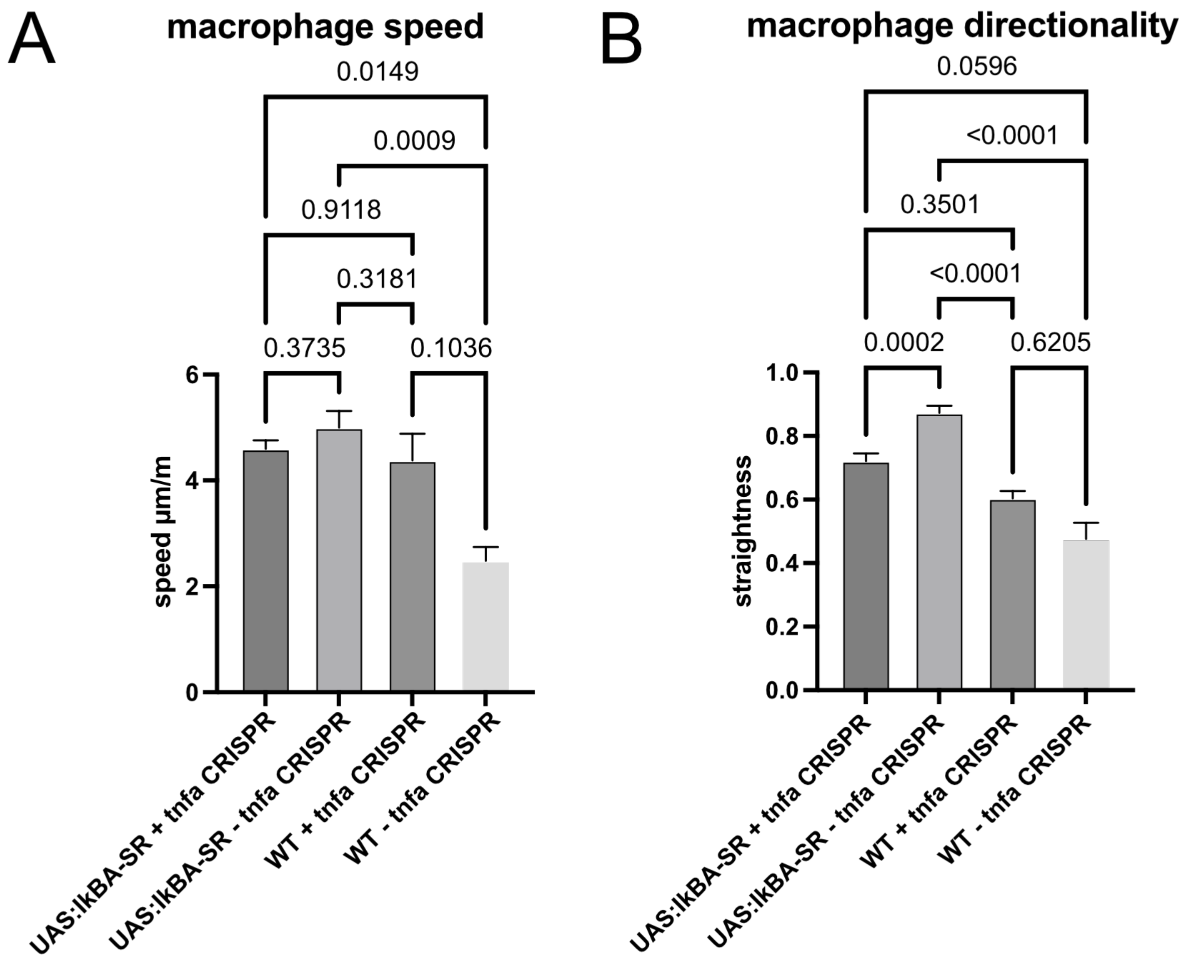

Supplementary Figure 10: quantification of macrophage speed and directionality in larvae with CRISPR/Cas9 mutagenesis of tnfa and macrophage over-expression of IKBA-SR. Macrophages were evaluated in uninjected wildtype larvae (WT/ -tnfa CRISPR), injected wildtype larvae (WT/ +tnfa CRISPR), uninjected transgenic mpeg:gal4; UAS:IKBASR larvae (IKBASR/ -tnfa CRISPR), injected transgenic mpeg:gal4; UAS:IKBASR larvae (IKBASR/ +tnfa CRISPR). Statistical tests were performed by 2-way ANOVA with Tukey's test for multiple comparisons with p-values shown for comparisons between conditions. Bar plots show mean values with bars representing standard error of the mean.

```
WT                TGTGCACCGTGTCCAACTGGGACGTTTTCAGACCGCAGTGGCT
tnfrsf1b gRNA     TGTGCATCGTGTCCAACTG--ACGTTTTCAGACCGCAGTGGCT

WT                TGTGCACCGTGTCCAACTGG-GACGTTTTCAGACCGCAGTGGCT
tnfrsf1b gRNA     TGTGCACCGTGTCCAACTGGAGACGTTTTCAGACCGCAGTGGCT

WT                TGTGCACCGTGTCCAACTGGGACGTTTTCAGACCGCAGTGGCT
tnfrsf1b gRNA     TGTGCACCGTG---------GACCGCAGTGGCT
```

Supplementary Figure 11: Representative sequences of the *tnfrsf1b* gene from larvae injected by Cas9 protein and gRNAs designed to *tnfrsf1b* exon 1. Alignments show differences (in red) between wildtype genome sequence (WT) and from injected larvae (tnfrsf1b gRNA). Deletions are shown by dashed lines.

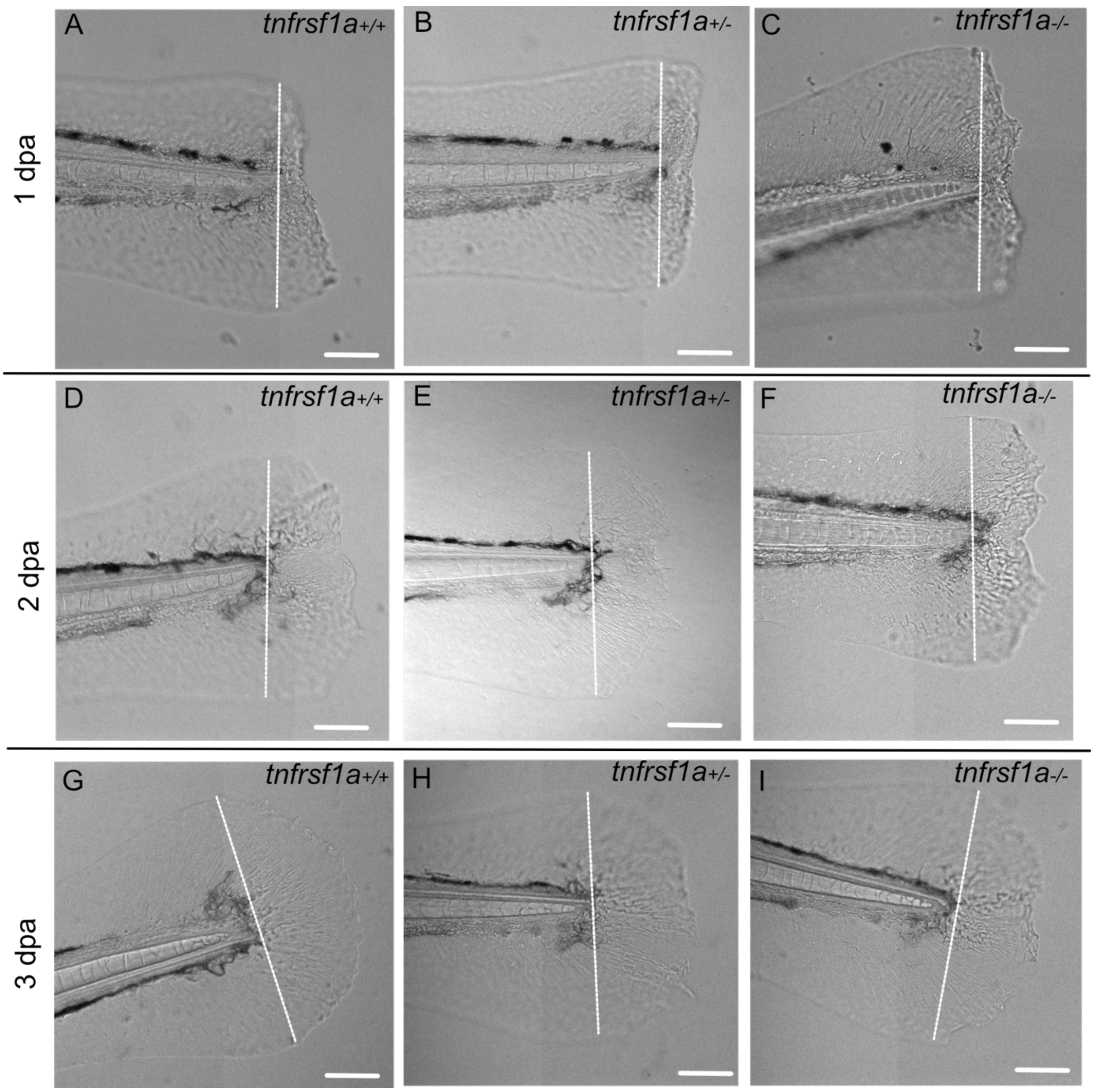

Supplementary Figure 12: tailfin regeneration is impaired in *tnfrsf1a*-/- mutants in a genotype-dependent manner. Regrowth of the fin fold in 4 dpf larvae was measured relative to the notochord (line) at 1 (A-C), 2, (D-F), 3 (G-I) days post amputation (dpa) in wildtype (*tnfrsf1a+/+,* A, D, G), heterozygote (*tnfrsf1a+/-,* B, E, H) and mutants (*tnfrsf1a-/-,* C, F, I).
Scale bars 100µm (A-I).

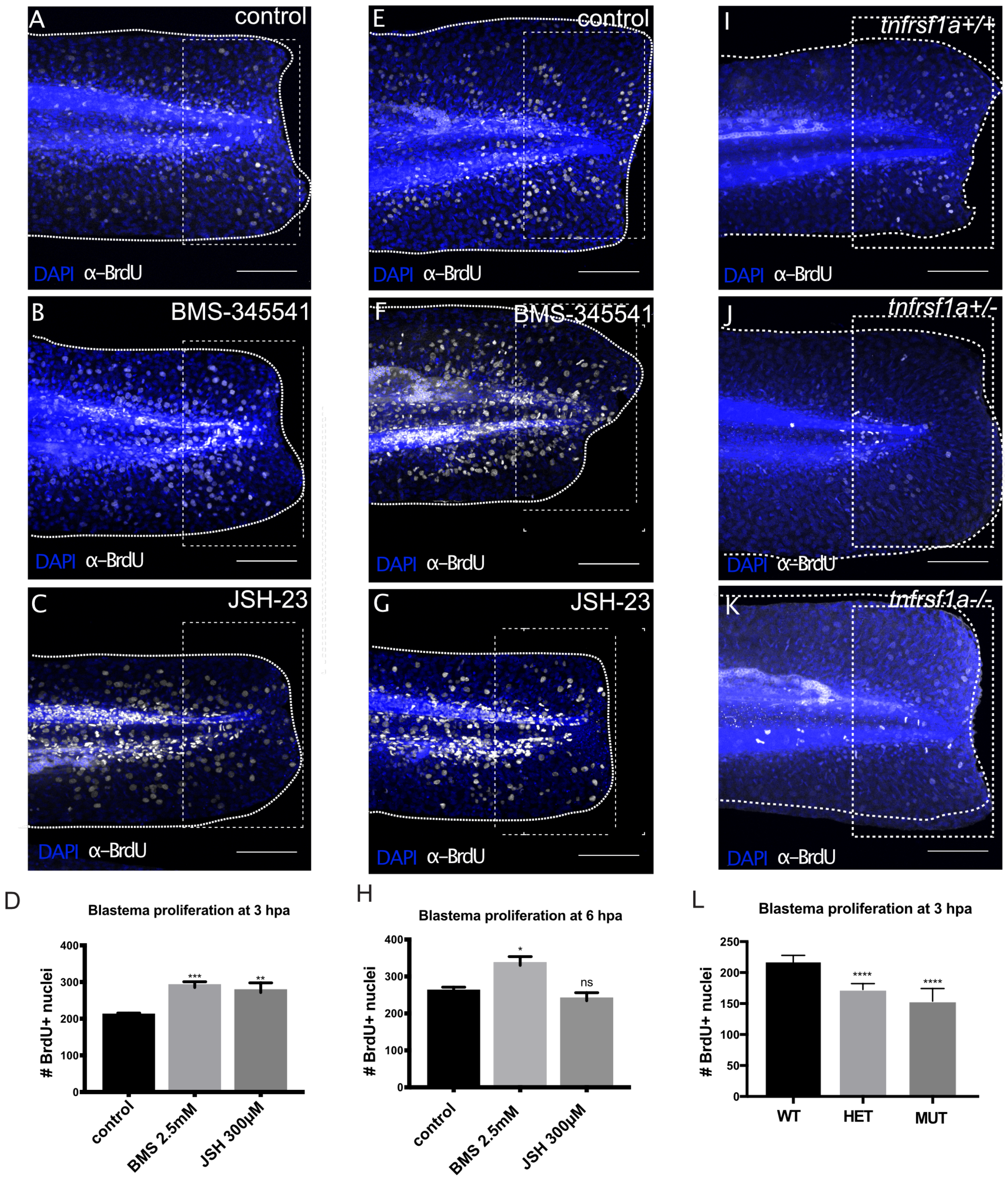

Supplementary Figure 13: proliferation is increased in regenerating tailfin in response to inhibition of NF-kB activity and in *tnfrsf1a-/-* mutants. Proliferating cells in the injured tail were detected at 3 (A-C) and 6 hpa (E-G) by BrdU incorporation in control larvae treated with DMSO (A, E) or larvae treated with BMS-354451 (B, F) or JSH-23 (C, G). BrdU+ cells were quantified at 3 hpa (D) and 6 hpa (H) and statistical differences between drug treated relative to controls shown. Proliferating cells in the injured tail of wildtype (I), heterozygote (J) and tnfrsf1a mutants (K) were detected at 3 hpa and plotted (L) with statistical differences relative to wildtype animals shown.

Statistical comparisons were performed by Student's unpaired t-test between genotypes at each stage. Significance is shown ($p<0.005$ ***, $p<0.01$ **, $p<0.05$ *, not significant n.s.). Bar plots show mean values with bars representing standard error of the mean.

Scale bars 100μm (A-I).

Supplementary Tables

Supplementary Table 1: quantification of macrophages recruited to injured tail over time hours post amputation (hpa)

time (hpa)	0	1	2	3	4
animal					
1	1	6	10	14	17
2	2	7	9	10	10
3	2	4	7	9	10
4	1	2	4	4	3
5	1	1	1	2	3
6	0	0	3	4	5

Supplementary Table 2: quantification of total macrophages and macrophages expressing NFKB:EGFP (GFP+) at injured tail over time hours post amputation (hpa)

	Total macrophages				
time (hpa)	0	1	2	3	4
animal					
1	0	1	1	2	2
2	0	5	5	7	8
3	0	1	6	6	6
mean	0	2.333333	4	5	5.333333
S.D.	0	2.309401	2.645751	2.645751	3.05505
S.E.M.	0	1.333333	1.527525	1.527525	1.763834

	GFP+ macrophages				
time (hpa)	0	1	2	3	4
animal					
1	0	0	1	1	1
2	0	0	0	3	3
3	0	1	3	3	3
mean	0	0.333333	1.333333	2.333333	2.333333
S.D.	0	0.57735	1.527525	1.154701	1.154701
S.E.M.	0	0.33333333	0.8819171	0.66666667	0.66666667

Supplementary Table 3: quantification of total macrophages and macrophages expressing tnfa:EGFP (GFP+) at injured tail over time hours post amputation (hpa)

	Total macrophages				
time (hpa)	0	1	2	3	4
animal					
1	1	6	10	14	17
2	2	7	9	10	10
3	2	4	7	9	10
4	1	2	4	4	3
5	1	1	1	2	3
6	0	0	3	4	5
mean	1.166667	3.333333	5.666667	7.166667	8
S.D.	0.752773	2.804758	3.559026	4.578937	5.440588
S.E.M.	0.434613	1.619328	2.054805	2.643651	3.141125

	GFP+ macrophages				
time (hpa)	0	1	2	3	4
animal					
1					
2	0	0	0	3	5
3	0	0	0	1	2
4	0	0	0	0	0
5	0	0	0	1	2
6	0	0	1	4	3
mean	0	0	0.2	1.8	2.4
S.D.	0	0	0.447214	1.643168	1.81659
S.E.M.	0	0	0.25819889	0.9486833	1.04880885

Supplementary Table 4: expression ratios for gene expression normalised to ef1a expression between uninjured control samples relative to injured samples calculated by the REST software. Significance is determined from the p value calculated by a pairwise fixed reallocation randomisation test.

Assay 1: uninjured vs injured						
		ef1a	ikbaa	ikbab	tnfa	il10
control	ratio	1	7.61	29.55	37.69	0.98
	p-value		0.00	0.04	0.00	0.98
BMS-345441	ratio		2.73	1.45	2247.16	158.63
	p-value		0.14	0.69	0.00	0.00
JSH-23	ratio		2.73	1.45	2247.16	158.63
	p-value		0.15	0.69	0.00	0.00
Assay 2: uninjured vs injured						
		ef1a	tnfb	il1b	il6	il8
control	ratio	1	5.86	9.52	41.76	1.70
	p-value		0.03	0.00	0.00	0.71
BMS-345441	ratio		1.78	0.62	1.47	0.99
	p-value		0.38	0.56	0.70	0.95
JSH-23	ratio		1.22	1.55	3.19	0.79
	p-value		0.89	0.85	0.57	0.86

Supplementary Table 5: quantification of macrophages expressing tnfa:egfp at the injured tail fin in control animals, JSH-23 treated or BMS-345541 treated larvae.

	Total macrophages - control						
time	cntrl2	cntr3	cntr4	cntrl5	cntrl6	Average	SD
t=0	3	3	2	2	1	2.167	0.548
t=1	8	5	3	2	1	4.333	2.550
t=2	10	8	5	2	4	6.667	3.701
t=3	11	10	5	3	5	8.167	4.817
t=4	11	11	4	4	6	9.000	5.857
t=5	12	11	4	4	5	10.167	8.585
t=6	12	11	6	6	4	12.500	12.498

	GFP+ macrophages - control						
time	cntrl2	cntr3	cntr4	cntrl5	cntrl6	Average	SD
t=0	1	1	1	1	1	1.000	0.000
t=1	1	1	1	1	1	1.000	0.000
t=2	1	1	1	1	2	1.200	0.447
t=3	1	2	1	2	5	2.200	1.643
t=4	6	3	1	3	4	3.400	1.817
t=5	11	6	1	6	4	5.600	3.647
t=6	11	9	3	8	4	7.000	3.391

	Ratio GFP+ macrophage/ total macrophage - control						
time	cntrl2	cntr3	cntr4	cntrl5	cntrl6	Average	SD
t=0	0.333	0.333	0.500	0.500	1.000	0.533	0.274
t=1	0.125	0.200	0.333	0.500	1.000	0.432	0.348
t=2	0.100	0.125	0.200	0.500	0.500	0.285	0.200
t=3	0.091	0.200	0.200	0.667	1.000	0.432	0.388
t=4	0.545	0.273	0.250	0.750	0.667	0.497	0.227
t=5	0.917	0.545	0.250	1.500	0.800	0.802	0.467
t=6	0.917	0.818	0.500	1.333	1.000	0.914	0.302

	Total macrophages – JSH-23						
time	animal 1	animal 2	animal 3	animal 4	animal 5	average	S.D.
t=0	3	1	1	4	3	2.400	1.342
t=1	5	2	1	6	8	4.400	2.881
t=2	9	2	2	8	11	6.400	4.159
t=3	11	4	2	10	11	7.600	4.278
t=4	12	4	2	11	13	8.400	5.030
t=5	13	4	2	13	13	9.000	5.523

	GFP+ macrophages – JSH-23						
time	animal 1	animal 2	animal 3	animal 4	animal 5	average	S.D.
t=0	2	1	1	3	3	2.000	1.000
t=1	4	2	1	5	8	4.000	2.739
t=2	9	2	2	8	11	6.400	4.159
t=3	11	4	2	10	11	7.600	4.278
t=4	12	4	2	11	13	8.400	5.030
t=5	13	4	2	13	13	9.000	5.523

	Ratio GFP+ macrophage/ total macrophage – JSH-23						
time	animal 1	animal 2	animal 3	animal 4	animal 5	average	S.D.
t=0	0.667	1	1	0.75	1	0.883	0.162
t=1	0.8	1	1	0.833	1	0.927	0.101
t=2	1	1	1	1	1	1.000	0.000
t=3	1	1	1	1	1	1.000	0.000
t=4	1	1	1	1	1	1.000	0.000
t=5	1	1	1	1	1	1.000	0.000

	Total macrophages – BMS-345541					
time	animal 1	animal 2	animal 3	animal 4	average	S.D.
t=0	3	2	2	2	2.250	0.500
t=1	6	3	9	4	5.500	2.646
t=2	8	3	10	5	6.500	3.109
t=3	7	4	7	6	6.000	1.414
t=4	9	6	9	9	8.250	1.500

	GFP+ macrophages – BMS-345541					
time	animal 1	animal 2	animal 3	animal 4	average	S.D.
t=0	3	2	1	1	1.750	0.957
t=1	6	3	8	4	5.250	2.217
t=2	8	3	8	5	6.000	2.449
t=3	7	4	5	6	5.500	1.291
t=4	9	10	7	9	8.750	1.258

	Ratio GFP+ macrophages/ total macrophages – BMS-345541					
time	animal 1	animal 2	animal 3	animal 4	average	S.D.
t=0	1.000	1.000	0.500	0.500	0.750	0.289
t=1	1.000	1.000	0.889	1.000	0.972	0.056
t=2	1.000	1.000	0.800	1.000	0.950	0.100
t=3	1.000	1.000	0.714	1.000	0.929	0.143
t=4	1.000	1.000	0.778	1.000	0.944	0.111

Supplementary Table 6: directionality and speed of individual macrophages responding to injury in control and NF-kB inhibitor treated larvae

		directionality	speed
condition	cell		
control	1	0.564	2.466
	2	0.983	4.252
	3	0.843	2.616
	4	0.080	2.034
	5	0.553	2.880
	6	0.172	2.454
	7	0.377	2.004
	8	0.514	2.865
	9	0.728	2.679
	10	0.292	6.144
average		0.511	3.039
S.D.		0.289	1.257

		directionality	speed
condition	cell		
JSH-23	1	0.751	3.449
	2	0.703	3.025
	3	0.846	2.844
	4	0.905	3.480
	5	0.953	3.230
	6	0.851	5.502
	7	0.900	5.217
	8	0.977	4.719
	9	0.576	3.626
	10	0.769	5.147
	11	0.813	4.187
	12	0.847	2.672
	13	0.966	1.601
average		0.835	3.746
S.D.		0.114	1.150

		directionality	speed
condition	cell		
BMS-345441	1	0.940	5.400
	2	0.900	3.300
	3	0.900	3.000
	4	0.900	4.380
	5	0.968	5.300
	6	0.974	4.150
	7	0.975	5.300
	8	0.858	4.300
	9	0.873	5.600
	10	0.885	3.500
	11	0.959	3.820
	12	0.817	4.300
	13	0.915	5.150
	14	0.960	4.000
	15	0.792	3.380
	16	0.952	3.110
	17	0.697	3.380
	18	0.715	3.000
	19	0.983	3.950
average		0.893	4.122
S.D.		0.085	0.870

Supplementary Table 7: quantification of macrophage directionality and speed in larvae with varying levels of tnfrsf1a activity in the presence of the TNFa inhibitor PTX. Macrophages were evaluated in wildtype (WT), heterozygote (HET), mutant (MUT) animals in control conditions or when treated with 70 µM PTX.

condition			Directionality			speed / µm min-1		
control	cell	genotype	WT	HET	MUT	WT	HET	MUT
	1		0.473	0.493	0.471	2.215	2.091	3.236
	2		0.582	0.334	0.318	3.822	3.002	3.123
	3		0.828	0.676	0.298	2.892	2.398	3.765
	4		0.908	0.415	0.632	3.730	3.303	3.194
	5		0.602	0.836	0.429	3.459	2.089	2.226
	6		0.879	0.872	0.894	3.408	4.130	3.915
	7		0.355	0.255	0.602	2.090	2.345	2.886
	8		0.363	0.719	0.574	2.702	3.610	2.781
	9		0.196			2.017		

condition			directionality		speed / µm min-1			
PTX	cell	genotype	WT	HET	MUT	WT	HET	MUT
	1		0.683	0.805	0.983	3.296	3.054	3.463
	2		0.495	0.844	0.820	2.593	1.798	3.555
	3		0.395	0.671	0.471	2.086	2.128	4.936
	4		0.316	0.804	0.839	2.850	2.628	5.982
	5		0.604	0.941	0.992	2.057	4.335	4.131
	6		0.371	0.720	0.824	1.906	2.892	7.524
	7		0.656	0.392	0.634	1.992	2.371	3.640
	8		0.722	0.640	0.906	4.123	3.028	1.829
	9		0.899	0.941	0.743	2.186	3.248	2.480
	10		0.450	0.849	0.703	2.029	3.244	6.476
	11		0.884	0.802	0.944	2.303	2.650	3.973
	12		0.545	0.925	0.793	3.420	3.376	2.767
	13		0.432	0.856	0.994	3.692	1.868	2.374
	14		0.348	0.669	0.659	3.617	1.276	2.275
	15		0.823		0.978	2.501		3.341
	16		0.429		0.917	3.138		2.308
	17		0.121			3.873		
	18		0.693					
	19		0.538					
	20		0.505					
	21		0.513					
	22		0.746					
	23		0.827					
	24		0.811					
	25		0.654					
	26		0.622					
	27		0.453					

	28		0.879					
	29		0.401					
	30		0.623					
	31		0.817					

Supplementary Table 8: quantification of macrophage directionality and speed in larvae expressing mpeg:gal4; UAS:IKBASR larvae in the absence (control) or presence of 70µM PTX.

condition	cell	directionality	speed/ um min-1
IKBASR/ control	1	0.745011	3.01659
	2	0.75412	4.985622
	3	0.983029	6.06534
	4	0.836375	4.387122
	5	0.680175	4.235946
	6	0.862162	2.638968
	7	0.937313	5.043354
	8	0.774902	5.172864
	9	0.5057	4.416204
	10	0.924078	5.074386
	11	0.994659	5.672268
	12	0.977721	6.31758
	13	0.922649	5.536908
	14	0.95668	5.350506
average		0.846755286	4.85097557
S.D.		0.1409118	1.04767963

condition	cell	directionality	speed/ um min-1
IKBASR / PTX	1	0.136691	3.542754
	2	0.846754	4.295688
	3	0.893496	3.11325
	4	0.704908	3.47121
	5	0.600569	3.620118
	6	0.638911	3.457914
	7	0.720141	2.972496
	8	0.592522	2.360706
	9	0.813012	2.93037
	10	0.726453	2.872848
	11	0.797569	2.838936
	12	0.78885	3.290028
	13	0.695612	3.755634
	14	0.822402	3.934998
	15	0.596027	3.511812
	16	0.804055	4.672434
	17	0.786215	4.634418
	18	0.702529	3.056574
	19	0.331091	2.959458
	20	0.478213	3.495804
	21	0.693821	3.01659
	22	0.583527	4.985622
	23	0.880525	6.06534
	24	0.777748	4.387122

	25	0.282146	4.235946
average		0.66775148	3.6591228
S.D.		0.18962492	0.834143

Supplementary Table 9: quantification of macrophage directionality and speed in larvae with CRISPR/Cas9 mutagenesis of tnfa and macrophage over-expression of IKBA-SR. Macrophages were evaluated in uninjected wildtype larvae (control/ uninjected), injected wildtype larvae (control/ TNFa CRISPR), uninjected transgenic mpeg:gal4; UAS:IKBASR larvae (IKBASR/ uninjected), injected transgenic mpeg:gal4; UAS:IKBASR larvae (IKBASR/ TNFa CRISPR) over a 4 hour time course.

		directionality	speed/ um min-1
condition	cell		
control/ uninjected	1	0.5640	0.0411
	2	0.9830	0.0709
	3	0.8430	0.0436
	4	0.0800	0.0339
	5	0.5530	0.0480
	6	0.1720	0.0409
	7	0.3770	0.0334
average		0.5103	0.0445
s.d.		0.3307	0.0127

		directionality	speed/ um min-1
condition	cell		
control/ TNFa CRISPR	1	0.5139	0.0478
	2	0.7282	0.0446
	3	0.2922	0.1024
	4	0.6576	0.0833
	5	0.8176	0.0842
	6	0.6345	0.0760
	7	0.6897	0.0763
	8	0.9060	0.0697
	9	0.6543	0.0600
	10	0.2688	0.0606
	11	0.4777	0.0748
	12	0.4633	0.0470
average		0.5920	0.0689
s.d.		0.1948	0.0175

		directionality	speed/ um min-1
condition	cell		
IKBASR/ uninjected	1	0.5060	0.0690
	2	0.8536	0.1112
	3	0.8746	0.1344
	4	0.8939	0.1154
	5	0.8415	0.0955
	6	0.9473	0.1219
	7	0.9461	0.0703
	8	0.9344	0.0610
	9	0.9346	0.0745
	10	0.7844	0.1012
	11	0.8786	0.0822
	12	0.9635	0.1323
	13	0.6932	0.0389
	14	0.9011	0.0894
	15	0.9075	0.0706
	16	0.8898	0.0570
	17	0.8354	0.0771
	18	0.9396	0.1397
	19	0.9647	0.1032
	20	0.9914	0.0853
	21	0.9441	0.0987
	22	0.7310	0.0966
	23	0.8084	0.0569
	24	0.8054	0.0529
	25	0.9494	0.0617
	26	0.9627	0.0852
	27	0.9427	0.0859
	28	0.9253	0.1135
	29	0.8675	0.0836
	30	0.9005	0.0758
	31	0.8109	0.0688
	32	0.9476	0.0674
	33	0.8391	0.0718
	34	0.9259	0.0615
	35	0.9555	0.0990
	36	0.8385	0.0922
	37	0.8756	0.0896
	38	0.9550	0.0810
average		0.8807	0.0861
s.d.		0.0924	0.0237

		directionality	speed/ um min-1
condition	cell		
IKABSR/ TNFa CRISPR	1	0.7483	0.0791
	2	0.7418	0.0545
	3	0.8953	0.0873
	4	0.5557	0.0561
	5	0.6799	0.0665
	6	0.6935	0.0703
	7	0.7452	0.1254
	8	0.5514	0.0852
	9	0.7235	0.0711
	10	0.8136	0.0553
	11	0.3429	0.0624
	12	0.5016	0.0993
	13	0.9541	0.1327
	14	0.4244	0.0672
	15	0.2254	0.0778
	16	0.6456	0.0614
	17	0.5876	0.0794
	18	0.8667	0.0812
	19	0.7026	0.0535
	20	0.8011	0.1054
	21	0.5879	0.0834
	22	0.6203	0.0630
	23	0.6509	0.0604
	24	0.5708	0.0592
	25	0.6413	0.0560
	26	0.8735	0.0392
	27	0.4097	0.0944
	28	0.5618	0.0531
	29	0.9236	0.1336
	30	0.8816	0.0783
	31	0.8292	0.0715
	32	0.9034	0.0942
	33	0.9650	0.1185
	34	0.9259	0.1086
	35	0.9555	0.0714
	36	0.8385	0.0727
average		0.7039	0.0786
s.d.		0.18581846	0.02357307

Supplementary Table 10: quantification of macrophage directionality and speed in larvae with CRISPR/Cas9 mutagenesis of *tnfrsf1b* and inhibition of NF-kB activity using BMS-345541. Macrophage movement was evaluated in uninjected wildtype larvae (control/ uninjected), injected wildtype larvae (control/ CRISPR), uninjected transgenic mpeg:gal4; UAS:IKBASR larvae (IKBASR/ uninjected), injected transgenic mpeg:gal4; UAS:IKBASR larvae (IKBASR/ TNFa CRISPR) over a 4 hour time course.

		directionality	speed/ um min-1
condition	cell		
control/ uninjected	1	0.557	2.58
	2	0.436	2.1
	3	0.319	1.44
	4	0.591	2.58
	5	0.57	3.06
	6	0.844	2.4
	7	0.425	2.1
	8	0.697	1.38
	9	0.469	1.32
	10	0.35	2.04
	11	0.589	0.54
	12	0.373	1.8
	13	0.411	2.04
	14	0.544	2.34
	15	0.688	0.66
average		0.5242	1.892
s.d.		0.1454	0.7095

		directionality	speed/ um min-1
condition	cell		
control/ tnfrsf1b CRISPR	1	0.65	1.08
	2	0.51	1.8
	3	0.72	1.08
	4	0.59	3.18
	5	0.677	3.36
	6	0.61	2.76
	7	0.76	2.16
	8	0.69	2.16
	9	0.539	1.26
	10	0.66	1.44
	11	0.52	2.1
	12	0.7	2.28
	13	0.68	0.36

	14	0.75	2.4
	15	0.71	2.58
	16	0.64	0.72
	17	0.67	1.86
average		0.6515	1.916
s.d.		0.0754	0.8369

		directionality	speed/ um min-1
condition	cell		
BMS-345541/ uninjected	1	0.901	3.82
	2	0.822	3.1
	3	0.866	4.5
	4	0.912	2.9
	5	0.958	3.7
	6	0.88	4
	7	0.872	3.3
	8	0.793	3.9
	9	0.79	2.6
	10	0.983	3.8
	11	0.989	4.1
	12	1	3.6
	13	0.939	3.2
	14	0.945	3.4
	15	0.785	3
	16	0.721	4
	17	0.803	3.6
average		0.8799	3.560
s.d.		0.08354	0.4957

		directionality	speed/ um min-1
condition	cell		
BMS-345541/ tnfrsf1b CRISPR	1	0.567	2.64
	2	0.895	2.7
	3	0.789	3.7
	4	0.794	3.36
	5	0.275	3.96
	6	0.835	3.62
	7	0.775	2.1
	8	0.782	2.4
	9	0.799	3.38
	10	0.898	1.74
	11	0.86	2.34
	12	0.876	3.74
	13	0.776	2.34

	14	0.609	3.18
	15	0.845	3.24
	16	0.626	2.26
average		0.7501	2.919
s.d.		0.1612	0.6830

Supplementary Movies

Movie S1: timelapsed movie of macrophages (magenta) expressing NFKB:EGFP (green) responding to tailfin injury (injury at bottom).
Timestamp in minutes

Movie S2: timelapsed movie of macrophages (red) expressing tnfa:egfp (green) responding to tailfin injury (injury at bottom).
Timestamp in hours:minutes

Movie S3: timelapsed movie of macrophages (red) in injured tailfin of animals (injury at bottom) expressing tnfa:egfp reporter (green) treated with 2.5 mM BMS-345541.

Movie S4: timelapsed movie of macrophages (red) in injured tailfin of animals (injury at bottom) expressing tnfa:egfp reporter (green) treated with 300 µM JSH-23.
Timestamp in hours: minutes

Movie S5: timelapsed movie of macrophages (red) in injured tailfin of animals (injury at bottom).
Timestamp in hours: minutes

Movie S6: timelapsed movie of macrophages (red) in injured tailfin of animals treated with 2.5 mM BMS-345541 (injury at bottom).
Timestamp in hours: minutes

Movie S7: timelapsed movie of macrophages (red) in injured tailfin of animals treated with 300 µM JSH-23 (injury at bottom).
Timestamp in hours: minutes

Movie S8: timelapsed movie of macrophages (red) in uninjured tailfin of control animals (tail orientated towards left).
Timestamp in hours: minutes

Movie S9: timelapsed movie of macrophages (red) in uninjured tailfin of animals treated with 2.5 mM BMS-345541 (tail orientated towards left).
Timestamp in hours: minutes

Movie S10: timelapsed movie of macrophages (magenta, tracks) migrating towards injured tailfin (injury at top) in a *tnfsfr1a* wildtype sibling.
Timestamp in hours: minutes

Movie S11: timelapsed movie of macrophages (magenta, tracks) migrating towards injured tailfin (injury at bottom) of a *tnfsfr1a* heterozygote animal.
Timestamp in hours: minutes

Movie S12: timelapsed movie of macrophages (magenta, tracks) towards injured tailfin (injury at bottom) of a *tnfsfr1a* mutant animal.
Timestamp in hours: minutes

Movie S13: timelapsed movie of macrophages (magenta) migrating towards injured tailfin (injury to right) of control fms:gal4; UAS:NsfB-mCherry animals (not expressing IKBASR).
Timestamp in hours: minutes

Movie S14: timelapsed movie of macrophages (magenta) migrating towards injured tailfin (injury to right) of transgenic fms:gal4; UAS:NsfB-mCherry; UAS:IKBASR animals.
Timestamp in hours: minutes

Movie S15: timelapsed movie of macrophages (magenta) migrating towards injured tailfin (injury at bottom) of transgenic fms:gal4; UAS:IKBASR CRISR control animals (not injected by tnfa gRNA/ Cas9).
Timestamp in hours: minutes

Movie S16: timelapsed movie of macrophages (magenta) migrating towards injured tailfin (injury at bottom) of transgenic fms:gal4; UAS:IKBASR animals injected by tnfa gRNA/ Cas9.
Timestamp in hours: minutes

Movie S17: timelapsed movie of macrophages (red) migrating towards injured tailfin (injury at bottom) of control uninjected animals.
Timestamp in hours: minutes

Movie S18: timelapsed movie of macrophages (magenta) migrating towards injured tailfin (injury at bottom) of control animals injected by Cas9 protein and gRNAs to tnfrsf1b.
Timestamp in hours: minutes

Movie S19: timelapsed movie of macrophages (magenta) migrating towards injured tailfin (injury at bottom) of uninjected larvae treated with BMS-345541.
Timestamp in hours: minutes

Movie S20: timelapsed movie of macrophages (magenta) migrating towards injured tailfin (injury at bottom) of animals injected by Cas9 protein and gRNAs to tnfrsf1b and treated with BMS-345541.
Timestamp in hours: minutes
